# Myeloid Zfhx3 deficiency protects against hypercapnia-induced suppression of host defense against influenza A virus

**DOI:** 10.1172/jci.insight.170316

**Published:** 2024-01-16

**Authors:** S. Marina Casalino-Matsuda, Fei Chen, Francisco J. Gonzalez-Gonzalez, Hiroaki Matsuda, Aisha Nair, Hiam Abdala-Valencia, G.R. Scott Budinger, Jin-Tang Dong, Greg J. Beitel, Peter H.S. Sporn

**Affiliations:** 1 Division of Pulmonary and Critical Care Medicine, Feinberg School of Medicine, Northwestern University, Chicago, Illinois, USA.; 2 Department of Physical Sciences and Engineering, Wilbur Wright College, Chicago, Illinois, USA.; 3 Research Service, Jesse Brown Veterans Affairs Medical Center, Chicago, Illinois, USA.; 4 Department of Human Cell Biology and Genetics, School of Medicine, Southern University of Science and Technology, Shenzhen, China.; 5 Department of Molecular Biosciences, Weinberg College of Arts and Sciences, Northwestern University, Evanston, Illinois, USA.

**Keywords:** Immunology, Pulmonology, Influenza, Innate immunity, Macrophages

## Abstract

Hypercapnia, elevation of the partial pressure of CO_2_ in blood and tissues, is a risk factor for mortality in patients with severe acute and chronic lung diseases. We previously showed that hypercapnia inhibits multiple macrophage and neutrophil antimicrobial functions and that elevated CO_2_ increases the mortality of bacterial and viral pneumonia in mice. Here, we show that normoxic hypercapnia downregulates innate immune and antiviral gene programs in alveolar macrophages (AMØs). We also show that zinc finger homeobox 3 (Zfhx3) — a mammalian ortholog of zfh2, which mediates hypercapnic immune suppression in *Drosophila* — is expressed in mouse and human macrophages. Deletion of Zfhx3 in the myeloid lineage blocked the suppressive effect of hypercapnia on immune gene expression in AMØs and decreased viral replication, inflammatory lung injury, and mortality in hypercapnic mice infected with influenza A virus. To our knowledge, our results establish Zfhx3 as the first known mammalian mediator of CO_2_ effects on immune gene expression and lay the basis for future studies to identify therapeutic targets to interrupt hypercapnic immunosuppression in patients with advanced lung disease.

## Abstract 2

<p>Myeloid lineage-specific deletion of <em>Zfhx3</em>&nbsp;decreases&nbsp;viral replication, inflammatory lung injury, and mortality in hypercapnic mice infected with influenza A virus.</p>

## Graphical abstract

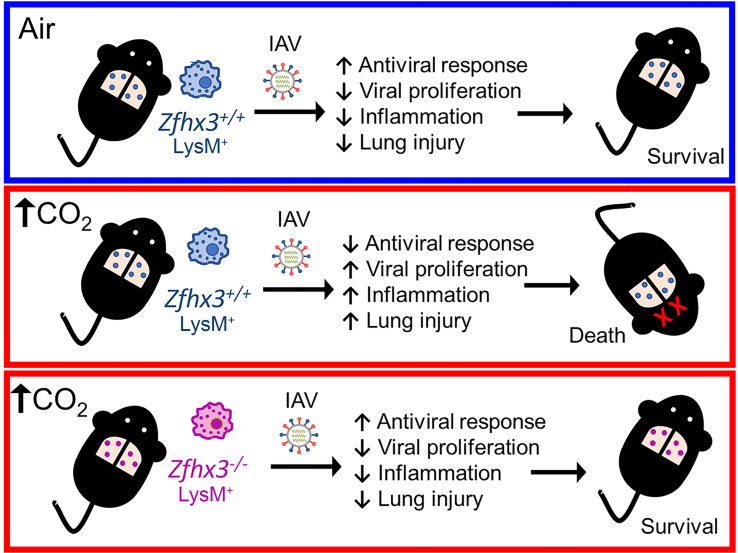

## Introduction

Hypercapnia, elevation of the partial pressure of CO_2_ (PCO_2_) in blood and tissue, commonly occurs in advanced chronic obstructive pulmonary disease (COPD) and in acute respiratory failure. Patients with severe COPD frequently develop bacterial and viral lung infections (1), including influenza (2–4), and hypercapnia is a risk factor for mortality in such individuals (5–9). Hypercapnia is also an independent risk factor for mortality in community-acquired pneumonia (10), adenoviral lung infections (11), and cystic fibrosis (12). Previously, we reported that hypercapnia suppresses transcription of multiple NF-κB–regulated innate immune genes required for host defense and inhibits phagocytosis in human, mouse, and *Drosophila* cells (13–15). We also showed that hypercapnia inhibits autophagy-mediated bacterial killing by macrophages (MØs) (16). Moreover, we found that hypercapnia increases mortality due to bacterial infections in both mice (17) and *Drosophila* (13). Recently, we showed that normoxic hypercapnia inhibits antiviral gene and protein expression and increases viral replication, lung injury, and mortality in mice infected with influenza A virus (IAV) (18). The suppressive effect of elevated CO_2_ on antiviral gene and protein expression and the hypercapnia-induced increase in IAV replication were particularly striking in lung MØs and were mediated by CO_2_-induced activation of Akt1 (18).

The similarity of hypercapnia’s effects in the *Drosophila* and mammalian systems suggested that elevated CO_2_ inhibits innate immune gene expression by conserved pathways. To identify putative molecular mediators of such pathways, we conducted a genome-wide RNA interference (RNAi) screen for genes whose expression was required for hypercapnic suppression of an antimicrobial peptide in cultured *Drosophila* cells. The screen identified multiple candidate CO_2_ mediators, the most potent of which was zfh2, a zinc finger homeobox transcription factor not previously known to have immunoregulatory function (19). Strikingly, mutant *Drosophila* deficient in zfh2 were protected against the CO_2_-induced increase in mortality from bacterial infection (19). To our knowledge, these results identified Zfh2 as the first known mediator of hypercapnic immune suppression in vivo. Like *Drosophila* Zfh2, its mammalian ortholog, zinc finger homeobox 3 (Zfhx3), also known as AT-binding transcription factor 2 (Atbf1), is a very large zinc-finger homeodomain transcription factor. Two isoforms, Zfhx3-A (MW 404 kD) and Zfhx3-B (MW 306 kD), are expressed as a result of alternative promoter usage combined with alternative splicing (20). Zfhx3 regulates neuronal differentiation (21), functions as a tumor suppressor (22), and sequence variants of the gene associated with atrial fibrillation (23). In the mouse, homozygous germline deletion of Zfhx3 is embryonic lethal, and heterozygosity results in partial fetal loss and high neonatal and preweaning mortality (24). Lung MØs are critical for resistance to influenza virus infection (25–28). This, along with our findings that hypercapnia downregulates MØ antiviral gene and protein expression (18) and that Zfh2 deficiency abrogates hypercapnic suppression of resistance to bacterial infection in *Drosophila* (19), led us to hypothesize that Zfhx3 might mediate the effects of elevated CO_2_ on lung MØs and host defense against IAV. Here we show that Zfhx3 is highly expressed in mouse and human MØs. We thus bred a mouse selectively lacking Zfhx3 in the myeloid lineage, which in the unstressed state grows without apparent phenotypic abnormalities. We now report that deletion of Zfhx3 abrogates hypercapnia-induced suppression of antiviral and innate immune gene expression in murine MØs and that myeloid Zfhx3 deficiency attenuates CO_2_-induced increases in viral replication, lung injury, and mortality in mice infected with IAV. Our results define Zfhx3 as the first known component, to our knowledge, of a CO_2_ response pathway affecting the immune system and host defense in a mammalian system.

## Results

### Zfhx3 is expressed in MØs and neutrophils.

**Figure 1:**
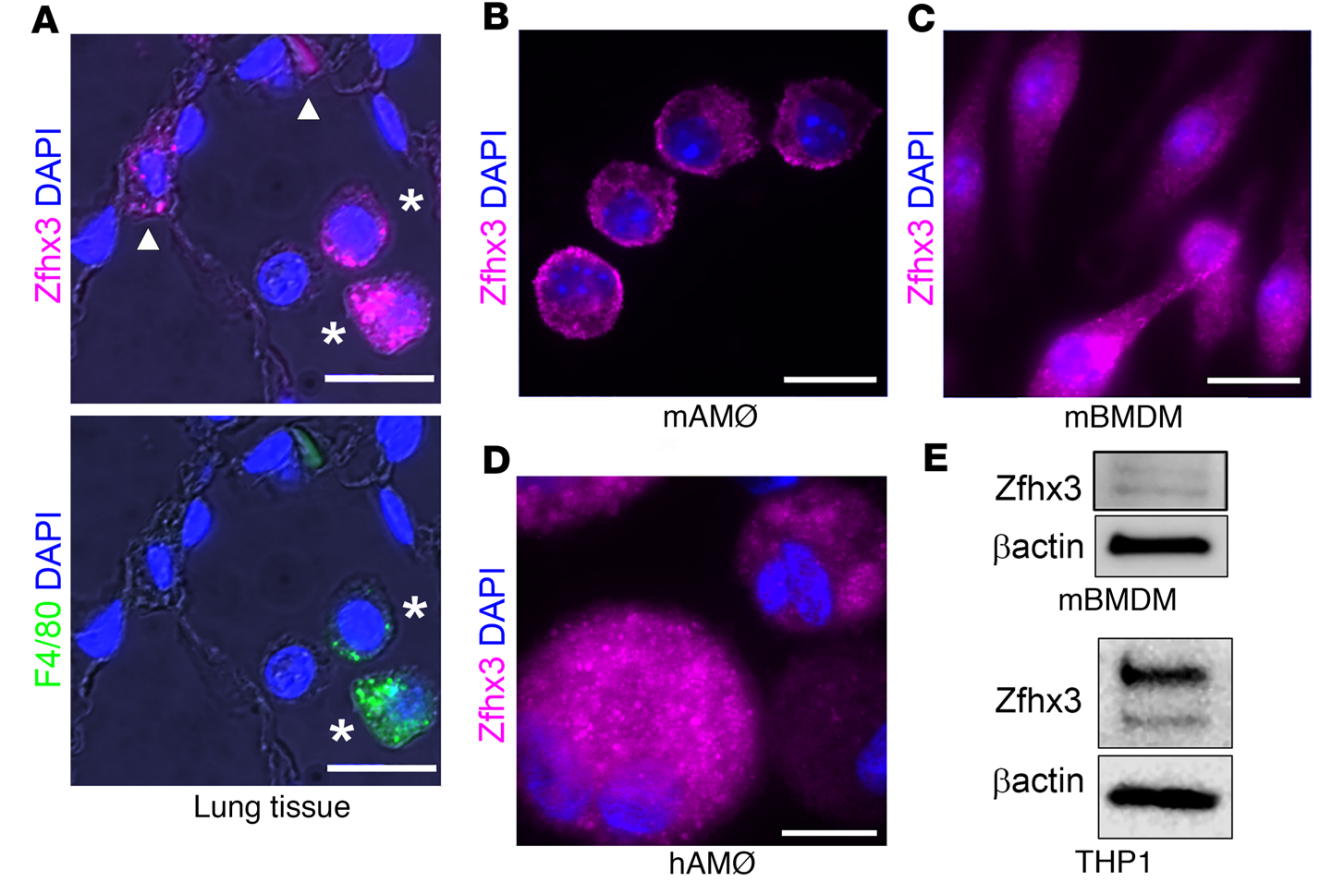
Zfhx3 is expressed in mouse and human alveolar macrophages. (**A**–**D**) Mouse lung tissue (**A**), mouse AMØs (**B**), cultured mouse BMDM (**C**), and human AMØs (**D**) were fixed and stained with specific anti-Zfhx3 antibody (magenta). Lung tissue in **A** was double stained with anti-F4/80 (green) to identify MØs (stars). In **A** (top panel), alveolar epithelial cells (arrow heads) also stain for Zfhx3. Nuclei were stained with DAPI (blue). Scale bars: 10 μm. (**E**) Immunoblot of mouse BMDM (top panel) and human THP1 MØs (bottom panel) for Zfhx3 and β-actin.

Having previously established Zfh2 as a bona fide mediator of hypercapnic immune suppression in *Drosophila* (19), here we determined whether its ortholog, Zfhx3 (Atbf1), is expressed in mammalian MØs and neutrophils. Using specific antibodies, we demonstrated the presence of Zfhx3 protein by immunofluorescence microscopy (Figure 1, A–D) and immunoblotting (Figure 1E), and we confirmed the presence of Zfhx3 protein in both mouse (Figure 1, A–C, and E) and human MØs (Figure 1, D and E). Human MØs and mouse BM-derived MØs (BMDM) both demonstrated a major band at ~400 kD, corresponding to Zfhx3-A, and a minor band at ~300 kD, corresponding to Zfhx3-B (Figure 1E). In addition, we confirmed expression of Zfhx3 mRNA in murine BMDM and neutrophils by quantitative PCR (qPCR) (Supplemental Figure 1, E and F; supplemental material available online with this article; https://doi.org/10.1172/jci.insight.170316DS1).

### Zfhx3 expression is markedly reduced in alveolar MØs and neutrophils but not in alveolar type 2 cells from Zfhx3^fl/fl^ LyzM^Cre^ mice.

To determine the role of Zfhx3 in the response to hypercapnia, we sought to study a mouse that was genetically deficient in Zfhx3. However, global homozygous Zfhx3 deficiency is a lethal mutation, and heterozygosity results in preweaning mortality (24). For this reason, and because our previous findings strongly implicate the MØ as a key cellular target of hypercapnic immunosuppression (18), we crossed a Zfhx3^fl/fl^ (24) with LysM^Cre^ mice (Lyz2^tm1[cre]Ifo^) to generate mice with homozygous Zfhx3 deficiency in the myeloid lineage. The myeloid Zfhx3^–/–^ mutant (referred to hereafter as Zfhx3^–/–^) is fully viable, grows normally, and exhibits no apparent phenotypic abnormalities in the unchallenged state. Immunofluorescence microscopy shows that, while AMØs obtained by bronchoalveolar lavage (BAL) and lung tissue from Zfhx3^fl/fl^ controls (referred to hereafter as Zfhx3^+/+^) stain strongly for Zfhx3, the protein is undetectable in AMØs from Zfhx3^–/–^ mice (Supplemental Figure 1, A and B). Likewise, Zfhx3 protein is highly expressed in BMDM from Zfhx3^+/+^ mice but is undetectable by immunofluorescence or immunoblot in BMDM from Zfhx3^–/–^ mice (Supplemental Figure 1, C and D). In addition, Zfhx3 mRNA is > 95% decreased by qPCR in BMDM and BM neutrophils from Zfhx3^–/–^ mice compared with Zfhx3^+/+^ controls (Supplemental Figure 1, E and F). Besides MØs and neutrophils, Zfhx3 is expressed in the lung in surfactant protein C^+^ (SPC^+^) alveolar type 2 (AT2) epithelial cells (Supplemental Figure 1B and Supplemental Figure 2A). Because a small percentage of lung epithelial cells express LysM and can be targeted by LysM-Cre constructs (29), we quantified Zfhx3 protein in SPC^+^ AT2 cells in lung tissue from Zfhx3^+/+^ and Zfhx3^–/–^ mice. This analysis showed a small but nonsignificant decrease in Zfhx3 expression, measured as corrected total cell fluorescence (CTCF) and expressed as arbitrary units (AU), in AT2 cells from Zfhx3^–/–^ as compared with Zfhx3^+/+^ mice (Supplemental Figure 2B). Notably, AT2 cells from Zfhx3^+/+^ and Zfhx3^–/–^ mice exhibited almost identical patterns of gene expression, as analyzed by RNA-Seq (Supplemental Figure 2C), consistent with the observation that Zfhx3 expression was not significantly altered in AT2 cells from Zfhx3^fl/fl^ LyzM^Cre^ mice. Taken together, these results indicate that LysM^Cre^-mediated Zfhx3 knockdown in the lung was indeed selective for the myeloid lineage, as intended.

### Hypercapnia downregulates innate immune and antiviral gene expression in alveolar MØs.

**Figure 2:**
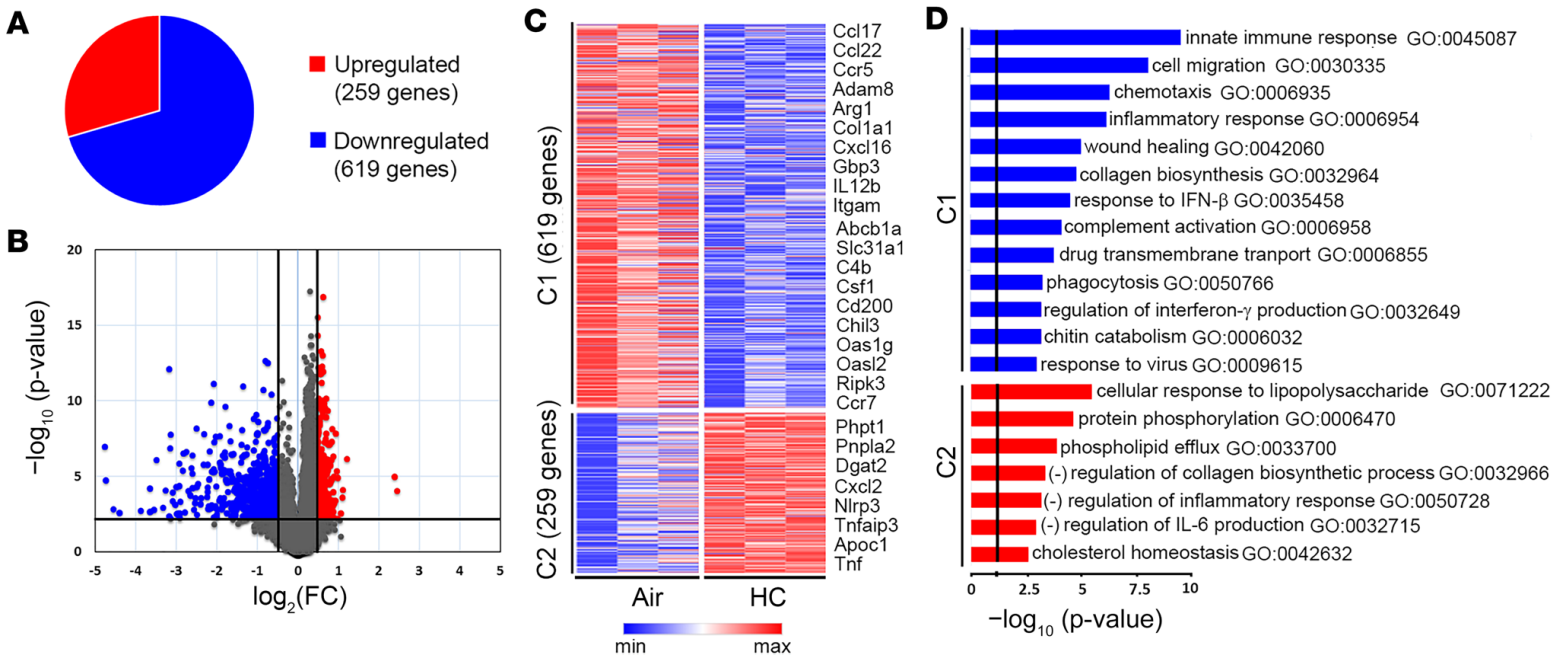
Hypercapnia alters gene expression in alveolar macrophages, resulting in downregulation of innate immune and antiviral pathways. AMØs isolated by flow cytometry from the lungs of mice exposed to ambient air or normoxic hypercapnia (10% CO_2_/21 % O_2_, HC) for 7 days were subjected to RNA-Seq. (**A**) Pie chart indicating proportion of genes downregulated or upregulated by hypercapnia. (**B**) Volcano plot showing statistical significance (−log_10_ [*P* value]) plotted against log_2_ fold change for hypercapnia versus air. Plot indicates significantly upregulated genes (log_2_ [fold change] ≥ +0.5, adjusted *P* <0.05) in red and downregulated genes (log_2_ [fold change] ≤ −0.5, adjusted *P* < 0.05) in blue. (**C**) K-means clustering of differentially expressed genes is presented as a heatmap of genes downregulated (cluster 1 [C1]) and upregulated (C2) in HC. (**D**) Bars indicate the top GO biological processes represented by genes downregulated (blue, C1) and upregulated (red, C2) genes in HC.

To assess the effect of elevated CO_2_ on global gene expression in Zfhx3^+/+^ AMØs, mice were exposed to ambient air or normoxic hypercapnia (HC, 10% CO_2_/21% O_2_) for 7 days, which we previously showed increases arterial PCO_2_ to ~75 mm Hg, as compared with ~40 mm Hg in air-breathing animals (17). After exposure to 10% CO_2_ or air as control for 7 days, lungs were harvested, and flow-sorted AMØs were subjected to bulk RNA-Seq. Global gene expression analysis revealed that hypercapnia significantly modified the expression of 878 genes ≥ ±1.4 fold (expressed as log_2_ [fold change]) with an adjusted *P* ≤ 0.05, including 619 downregulated and 259 upregulated genes (Figure 2A). The downregulated transcripts correspond to 2.8%, and the upregulated transcripts to 1.2% of the 22,000 murine protein-coding genes, indicating that hypercapnia-induced gene regulation is highly selective. The volcano plot in Figure 2B shows that the fold change for a large proportion of the downregulated genes was much greater than that for the upregulated genes. Differential gene expression was consistent in AMØs from replicate mice, as represented in the heatmap with K-means clustering (Figure 2C).

Gene Ontology (GO) analysis shows that hypercapnia downregulated multiple processes related to innate immunity and response to viral infection in AMØs (Figure 2D and Supplemental Figure 3A). Specific genes whose change in expression maps to antiviral processes downregulated by hypercapnia include Ripk3 (30), Msr1 (31), Cxcl17 (32), C1q (33), C4a (34), C4b (35), Cfh (36), Ch25h (37), Ccl17 (38), Cd200 (39), Gbp6 (40), Iigp1 (41), Pyhin1 (42), and the IFN-stimulated antiviral effector Oasl2 (43). Additional innate immune response genes downregulated by elevated CO_2_ relate to inflammatory responses (Adam8, Ccl22, Ccr5, Ccr7, Irg1, Ptgs2), chemotaxis (Ccl9, Cxcl15, Cxcl16, C3ar1), complement activation (Cfh, Hc, Serping1), and phagocytosis (Mfge8, Sftpa1, Slc11a1). Other hypercapnia-regulated genes map to processes downregulated by elevated CO_2_ that could also influence outcomes of infection; these include cell migration (Csf1, Cyr61, Hbegf, Mmp14, Pdgfr), wound healing (Dcbld2, Hpse, Mmp12, Mustn1, Pdgfb, Pdgfra, Slc11a1, Sparc, Timp1), collagen biosynthesis (Arg1, Col1a1, Col3a1, Serpinh1), and chitin catabolism (Chil3 and Chil4). Notable among the GO biological processes upregulated by hypercapnia are negative regulation of inflammation (Nlrp3, Nt5e, Tnfaip3, Zfp36) and of IL-6 (Tnf, Tnfaip3, Zc3h12a) (Figure 2D and Supplemental Figure 3B). Supplemental Figure 3, A and B, shows that many CO_2_-regulated genes map to gene networks shared by more than 1 GO process downregulated or upregulated by hypercapnia. In summary, the changes in AMØ gene expression resulting from exposure to elevated CO_2_ suggest mechanisms by which hypercapnia would be expected to increase susceptibility to and/or worsen the outcome of infections due to viruses and other pathogens.

In contrast to the major hypercapnia-induced changes in AMØ gene expression described above, there was no difference in global gene expression between AT2 cells isolated from Zfhx3^+/+^ mice exposed to 10% CO_2_/21% O_2_ for 7 days and those isolated from air-breathing controls (Supplemental Figure 2D). These data replicate our findings in WT mice (44). Thus, unlike AMØs, under these conditions, AT2 cells are not a primary target of elevated CO_2_ at the level of gene transcription, at least in the otherwise unchallenged state.

### Myeloid Zfhx3–deficient adult mice appear healthy and tolerate exposure to normoxic hypercapnia without illness or lung injury.

To assess whether elevated CO_2_ causes lung injury in control and myeloid Zfhx3–deficient animals, Zfhx3^+/+^ and Zfhx3^–/–^ mice were exposed to 10% CO_2_/21% O_2_, or ambient air as control, for 7 days before being sacrificed for examination of lung histology. The lungs of both air- and CO_2_-exposed Zfhx3^+/+^ and Zfhx3^–/–^ mice appeared histologically normal (Supplemental Figure 4A), and blinded scoring revealed no evidence of histopathologic lung injury in any of the groups (Supplemental Figure 4B). In addition, Zfhx3^+/+^ and Zfhx3^–/–^ mice gained weight similarly during exposure to ambient air or 10% CO_2_/21 % O_2_ for 21 days (Supplemental Figure 4C), and all animals survived these conditions (Supplemental Figure 4D).

### Zfhx3 deficiency abrogates hypercapnia-induced changes in expression of innate immune and inflammatory pathway genes in alveolar MØs.

**Figure 3:**
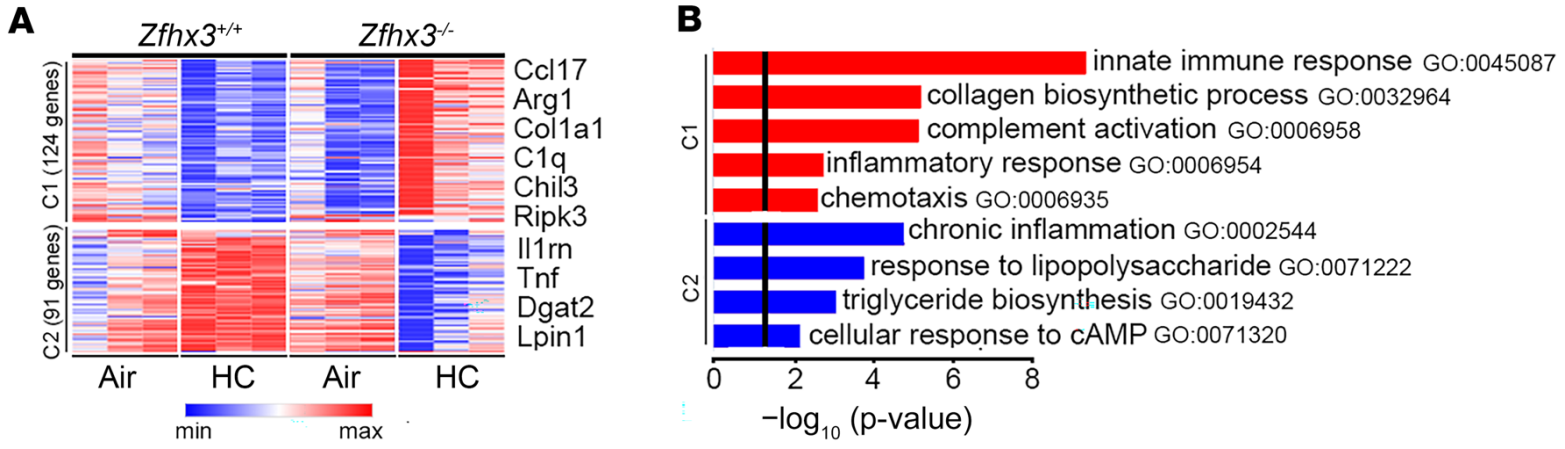
Zfhx3 deficiency abrogates hypercapnia-induced changes in expression of innate immune and inflammatory pathway genes in alveolar macrophages. AMØs isolated by flow cytometry from the lungs of Zfhx3^+/+^ and Zfhx3^–/–^ mice exposed to ambient air or normoxic hypercapnia (10% CO_2_/21% O_2_, HC) for 7 days were subjected to RNA-Seq. (**A**) K-means clustering of differentially expressed genes is presented as a heatmap of genes downregulated by hypercapnia in AMØs from Zfhx3^+/+^ mice but not those from Zfhx3^–/–^ mice (cluster 1 [C1]) and genes upregulated by hypercapnia in AMØs from Zfhx3^+/+^ mice but not those from Zfhx3^–/–^ mice (cluster 2, C2). (**B**) Bars indicate the top GO biological processes represented by genes in C1 (red) and C2 (blue).

Previously, we showed that knockdown of zfh2 blocked hypercapnic suppression of multiple antimicrobial peptides in *Drosophila* (19). Here, we were interested in determining the effect of myeloid Zfhx3 deficiency on hypercapnia-induced alterations in expression of immune, inflammatory, and other genes. Thus, we exposed Zfhx3^–/–^ and Zfhx3^+/+^ mice to normoxic hypercapnia (10% CO_2_/21% O_2_) or ambient air for 7 days, flow sorted AMØs, and subject them to RNA-Seq. The heatmap in Figure 3A shows that Zfhx3 deficiency reversed CO_2_-induced gene regulation of 124 genes whose expression was decreased by hypercapnia (cluster 1 [C1]) and 91 genes whose expression was increased by hypercapnia (C2) in AMØs from Zfhx3^+/+^ mice. GO analysis shows that the biologic processes upregulated under hypercapnic conditions in Zfhx3^–/–^ as compared with Zfhx3^+/+^ AMØs include innate immune and inflammatory responses, complement activation, and chemotaxis (Figure 3B). Many genes whose expression was suppressed by hypercapnia in Zfhx3^+/+^ AMØs, but not in Zfhx3^–/–^ AMØs, map to these processes and have established roles in antiviral host defense, including Ripk3 (30), Msr1 (31), Cxcl17 (32), C1q (33), C4a (34), C4b (35), Cfh (36), Ch25h (37), Ccl17 (38), and Cd200 (39). Biologic processes downregulated by hypercapnia in Zfhx3^–/–^ as compared with Zfhx3^+/+^ AMØs include response to LPS and cellular response to cAMP, involving the genes Ccrn4l, Il1rn, Nfkbia, Tnf, Egr3, and Gpd1. Multiple genes that were differentially expressed in Zfhx3^–/–^ as compared with Zfhx3^+/+^ AMØs under hypercapnic conditions map to more than 1 process (Supplemental Figure 5, A and B). The foregoing results indicate that Zfhx3 deficiency protects AMØs from CO_2_-induced changes in expression of specific sets of genes in a manner that would be expected to prevent or attenuate hypercapnic immunosuppression.

### Myeloid Zfhx3 deficiency protects against hypercapnia-induced increases in lung injury and mortality in mice infected with IAV.

**Figure 4:**
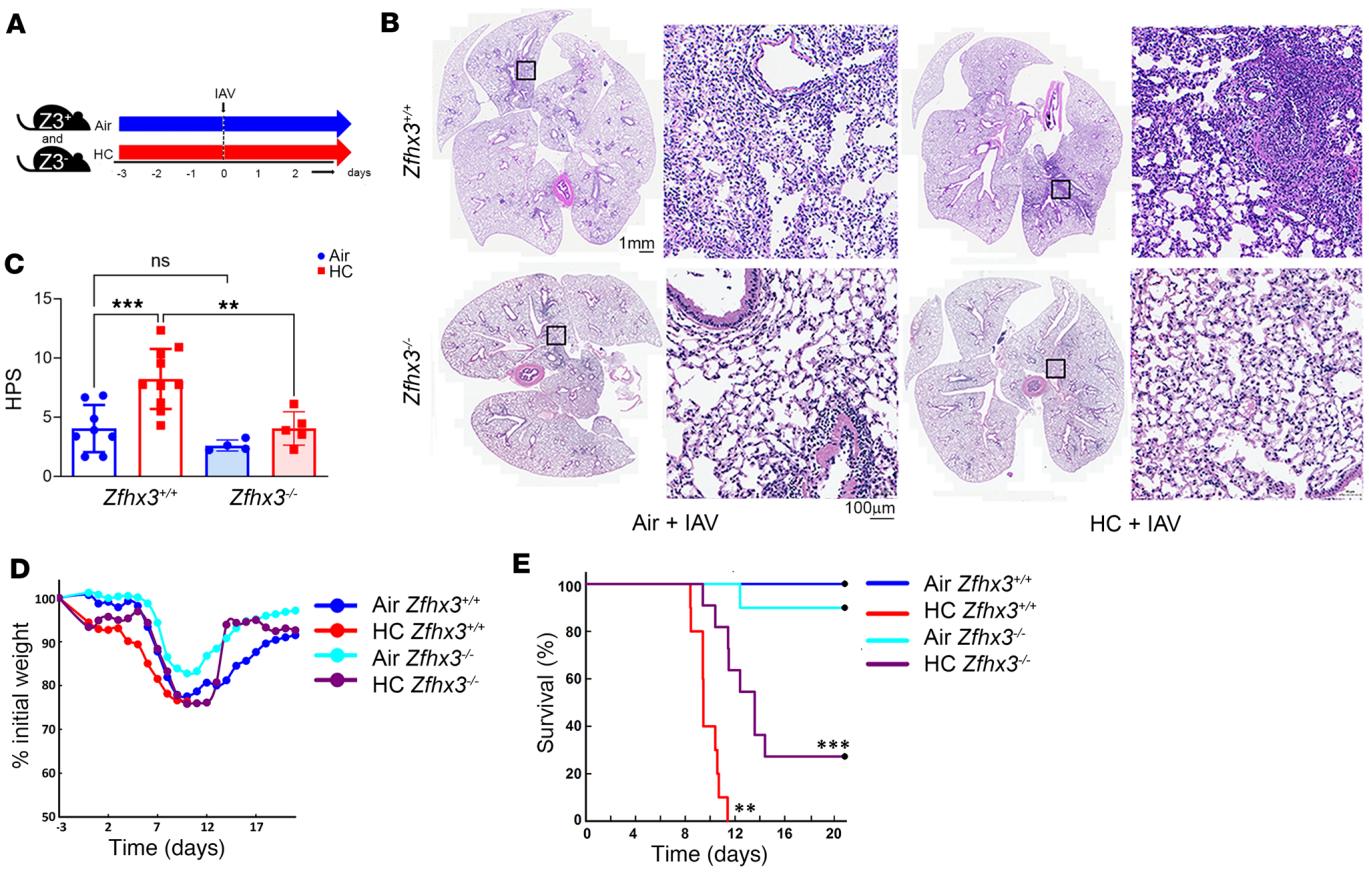
Myeloid *Zfhx3* deficiency protects against hypercapnia-induced increases in lung injury and mortality in IAV-infected mice. (**A**–**E**) Zfhx3^+/+^ (Z3^+^) and Zfhx3^–/–^ (Z3^–^) mice were preexposed to normoxic hypercapnia (10% CO_2_/21% O_2_, HC) for 3 days, or air as control, before being infected intratracheally with IAV (A/WSN/33) (**A**) at 30 (**B** and **C**) or 3 (**D** and **E**) pfu per animal; *n* = 6–10 per group. Lungs from IAV-infected mice harvested 4 dpi were sectioned and stained with H&E, and montage images of whole lung sections (**B**) were assessed to determine histopathologic scores (HPS) for lung injury (**C**) analyzed by 1-way ANOVA plus Sidak’s multiple-comparison test; **P < 0.01, ***P < 0.001. Body weight changes over time (**D**) and Kaplan-Meier plot of survival (**E**) after infection with 3 pfu IAV were analyzed by log-rank test. **P < 0.05 versus Air Zfhx3^+/+^, ***P < 0.05 versus HC Zfhx3^+/+^.

To determine the importance of Zfhx3 in CO_2_-induced suppression of antiviral host defense, we infected Zfhx3^–/–^ and Zfhx3^+/+^ mice breathing ambient air or 10% CO_2_/21% O_2_ with IAV. To minimize potential confounding effects of acidosis associated with hypercapnia, mice were preexposed to normoxic hypercapnia for 3 days prior to IAV infection. We previously showed that this preexposure time allows for maximal renal compensation of respiratory acidosis, resulting in an arterial pH of ~7.3, close to that of air-breathing controls (17). Mice were then inoculated with IAV (strain A/WSN/1933) at 3 or 30 pfu and maintained in air or 10% CO_2_/21% O_2_ until sacrifice at 4 or 7 days postinfection (dpi), or monitored until death or recovery (Figure 4A). As previously reported in WT mice (18), Zfhx3^+/+^ mice breathing 10% CO_2_ experienced greater histologic lung injury than air-breathing mice following infection with IAV at both 30 (Figure 4, B and C) and 3 (Supplemental Figure 6A) pfu per animal. By contrast, Zfhx3^–/–^ mice were protected against the increase in IAV-induced lung injury caused by hypercapnia, as shown in representative images (Figure 4B and Supplemental Figure 6A), and by histopathologic score (HPS) (Figure 4C). We assessed the effect of myeloid Zfhx3 deficiency on the mortality of IAV infection in mice inoculated with 3 pfu per animal. At this inoculum, all air-breathing Zfhx3^+/+^ mice survived (Figure 4E), while those exposed to hypercapnia lost more weight (Figure 4D) and all died by 11 dpi (Figure 4E). Similar to Zfhx3^+/+^ mice, among air-breathing Zfhx3^–/–^ mice, all but 1 survived IAV infection (Figure 4E). On the other hand, when exposed to hypercapnia and infected with IAV, Zfhx3^–/–^ mice survived longer than Zfhx3^+/+^ mice, and mortality in the Zfhx3^–/–^ mice was reduced to 73%, as compared with 100% in Zfhx3^+/+^ mice (Figure 4E). Interestingly, 10% CO_2_-exposed Zfhx3^+/+^ and Zfhx3^–/–^ mice lost a similar amount of weight after IAV infection (Figure 4D), suggesting that the protective effect of myeloid Zfhx3 deficiency on the outcome of IAV infection in the setting of hypercapnia was not due to improved intake of food and water in Zfhx3^–/–^ mice. Taken together, these results establish Zfhx3 as a bona fide mediator of CO_2_-induced immunosuppression in mice.

### Myeloid Zfhx3 deficiency prevents hypercapnia-induced increases in viral replication and suppression of antiviral gene and protein expression in IAV-infected mice.

**Figure 5:**
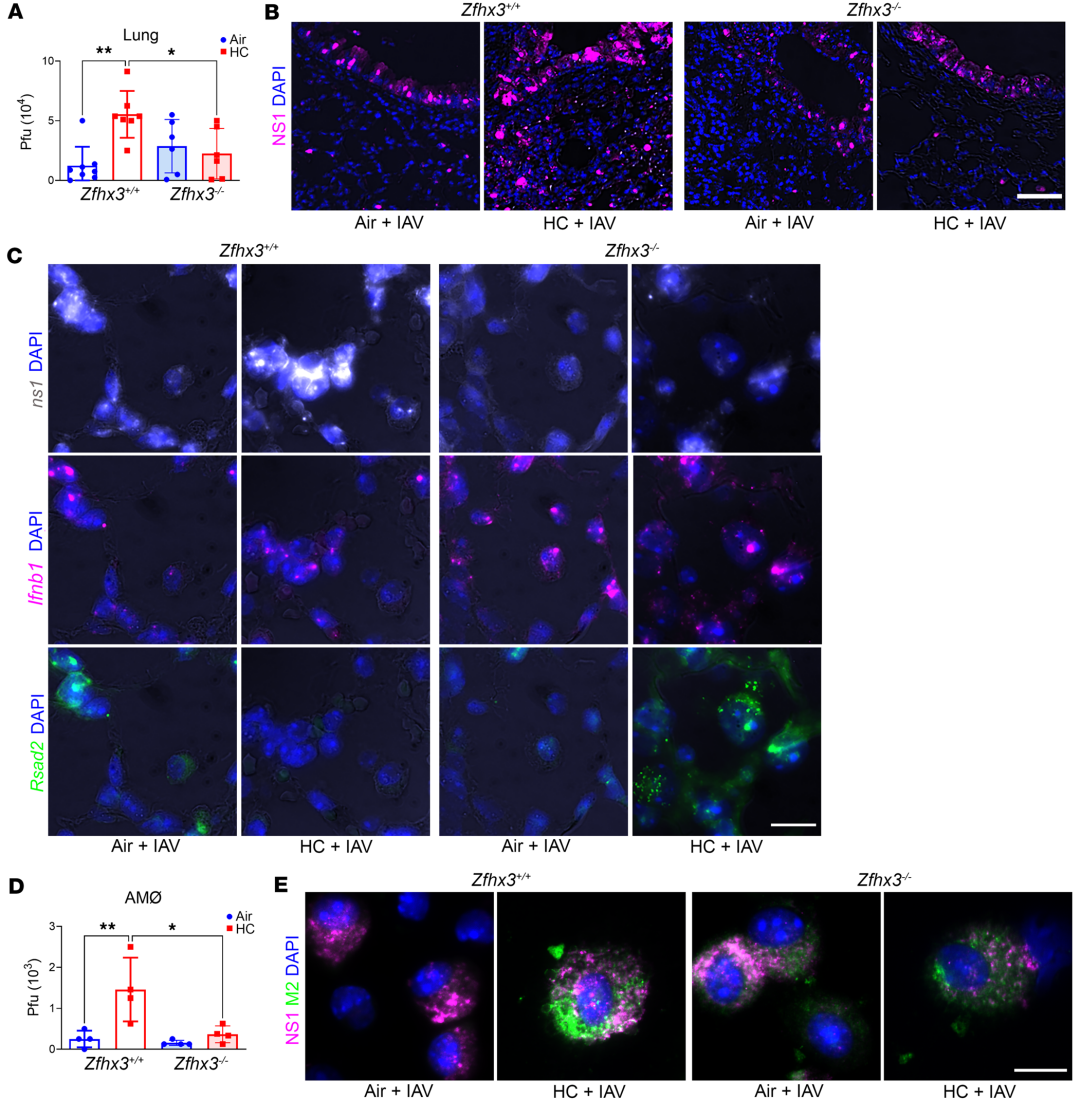
Myeloid *Zfhx3* deficiency prevents hypercapnia-induced increases in viral replication and suppression of antiviral gene and protein expression in IAV-infected mice. (**A**–**E**) Zfhx3^+/+^ and Zfhx3^–/–^ mice were preexposed to normoxic hypercapnia (10% CO_2_/21% O_2_, HC) for 3 days, or air as control, before being infected intratracheally with 30 (**A**, **B**, **D**, and **E**) or 300 (**C**) pfu IAV (A/WSN/33) per animal; *n* = 6–10 per group. Viral titers in homogenized lung tissue determined by plaque assay at 4 dpi (**A**). Expression of viral NS1 protein (magenta) assessed in lung tissue sections from mice sacrificed 4 dpi (**B**). Ifn1b, Rsad2, and viral ns1 transcript expression in lung tissue sections from mice infected with IAV 300 pfu 1 dpi was detected by RNAscope (**C**). AMØs from IAV-infected mice were obtained by BAL 1 dpi and cultured under normocapnic (5% CO_2_/95% air, NC) or hypercapnic (15% CO_2_/21% O_2_/64% N_2_, HC) conditions for 18 hours, after which viral titers in culture supernatants were determined by plaque assay (**D**) or assessed for viral NS1 (magenta) and M2 (green) protein expression by immunofluorescence microscopy (**E**). Nuclei were stained with DAPI (blue) (**B**, **C**, and **E**). In **A** and **D**, differences were analyzed by 1-way ANOVA plus Sidak’s multiple-comparison test; *P < 0.05, **P < 0.01. Scale bars: 50 μm (**B**) and 10 μm (**C** and **E**).

To understand mechanisms by which myeloid Zfhx3 deficiency protects against the adverse effect of hypercapnia on IAV-induced lung injury and mortality, we assessed viral loads and expression of viral and antiviral genes and proteins in lung tissue and AMØs from IAV-infected Zfhx3^+/+^ and Zfhx3^–/–^ mice. First, we found that, while exposure to 10% CO_2_ increased the amount of live virus recoverable from the lungs of Zfhx3^+/+^ mice 4 dpi by more than 2-fold, the hypercapnia-induced increase in viral load was blocked in Zfhx3^–/–^ mice (Figure 5A). Likewise, 10% CO_2_ exposure increased expression of the viral proteins NS1 and M2 in the lungs of IAV-infected Zfhx3^+/+^ mice, but this effect of hypercapnia was blocked in Zfhx3^–/–^ mice (Figure 5B and Supplemental Figure 6, B and D). Similarly, hypercapnia increased ns1 mRNA expression in lung tissue of Zfhx3^+/+^ mice, as assessed by RNAscope, and this was attenuated in the lungs of CO_2_-exposed Zfhx3^–/–^ mice (Figure 5C). Conversely, exposure to hypercapnia blunted or suppressed expression of mRNA and/or protein for Ifnb1 and the IFN-stimulated antiviral effectors Rsad2 and Oas1 in IAV-infected Zfhx3^+/+^ mice, whereas mRNA and/or protein expression of these antiviral factors was increased in the lungs of CO_2_-exposed Zfhx3^–/–^ mice (Figure 5C and Supplemental Figure 6, C and D).

To determine the effects of myeloid Zfhx3 deficiency on the response of lung MØs specifically, we studied AMØs isolated from Zfhx3^+/+^ and Zfhx3^–/–^ mice exposed to 10% CO_2_ or ambient air and infected with IAV (300 pfu), as described above. AMØs were obtained by BAL 1 dpi, washed, and cultured under normocapnia (NC, 5% CO_2_/95% air) or hypercapnia (HC, 15% CO_2_/21% O_2_/64% N_2_) for 18 h prior to analysis. Figure 5D shows that exposure of Zfhx3^+/+^ AMØs to hypercapnia in vivo and in vitro, as compared with air in vivo and NC in vitro, resulted in a 6-fold increase in recovery of viable virus, strongly suggesting active viral replication in these cells in the setting of elevated CO_2_. As in the homogenized lung, the hypercapnia-induced increase in viral load was almost fully blocked in AMØs from Zfhx3^–/–^ mice (Figure 5D). Also mirroring the finding in lung tissue, hypercapnia increased expression of viral NS1 and M2 protein (Figure 5E) and ns1 mRNA (Supplemental Figure 7) and reduced Rsad2 mRNA expression (Supplemental Figure 7) in AMØs from Zfhx3^+/+^ mice. These effects of elevated CO_2_ were blocked in AMØs from Zfhx3^–/–^ mice (Figure 5E and Supplemental Figure 7). Myeloid Zfhx3 deficiency also abrogated the increase of viral proteins NP, NS1, and M2 (Supplemental Figure 8, A–D) and suppression of antiviral protein expression of Oas1 and Rsad2 (Supplemental Figure 8, C and D) caused by hypercapnia in IAV-infected mouse BMDM.

Exposure of mice to elevated CO_2_ increased viral protein expression not only in AMØs but also in lung parenchymal cells, including AT2 and bronchial epithelial cells, as shown in Figure 5B and Supplemental Figure 6, B and D, and in our previous report (18). Of note, myeloid Zfhx3 deficiency not only blocked the hypercapnia-induced increase in expression of viral gene products and suppression of host antiviral factors in MØs, but it also blunted the increase in viral NS1 and M2 and the decrease in the antiviral effector Rsad2 in airway and alveolar epithelial cells of CO_2_-exposed mice (Figure 5, B and C, and Supplemental Figure 6, B and D). This suggests that myeloid Zfhx3 deficiency protects against hypercapnic suppression of antiviral host defense in vivo not only by enhancing the resistance of MØs to viral infection, but also by reducing viral infection and/or replication in lung epithelial cells.

## Discussion

In the present work, we show that the zinc finger homeodomain transcription factor Zfhx3, which was not previously known to regulate immune function or responses to CO_2_, is highly expressed in mouse and human MØs. We document that exposure of mice to 10% CO_2_ alters gene expression in AMØs, resulting in selective downregulation of innate immune and antiviral gene programs, and we show that KO of Zfhx3 in the myeloid lineage prevents hypercapnic downregulation of innate immune pathways and also blocks CO_2_-induced changes in other gene programs. Moreover, we found that myeloid Zfhx3^–/–^ mice were protected from hypercapnia-induced increases in viral replication, inflammatory lung injury, and mortality following IAV infection. Myeloid Zfhx3 deficiency blocked hypercapnic suppression of IFN-β and antiviral effector gene and protein expression as well as the CO_2_-induced increase in viral gene and protein expression caused by hypercapnia in IAV-infected Zfhx3^+/+^ mice. To our knowledge, these findings establish Zfhx3 as the first known mediator of CO_2_-induced immunosuppression in a mammalian system.

Exposure of mice to normoxic hypercapnia downregulated AMØ expression of genes involved in multiple GO processes that are likely relevant to host defense against IAV, including innate immune response, cell migration, chemotaxis, inflammatory response, response to IFN-β, complement activation, regulation of IFN-γ production, and response to virus. Specific genes with documented roles in host defense against influenza and other viral infections that were downregulated in AMØs include Ripk3 (30), Msr1 (31), Cxcl17 (32), C1q (33), C4a (34), C4b (35), Cfh (36), Ch25h (37), Ccl17 (38), Cd200 (39), Gbp6 (40), Iigp1 (41), Pyhin1 (42), and the IFN-stimulated antiviral effector Oasl2 (43). Myeloid Zfhx3 deficiency abrogated hypercapnic downregulation of GO processes in AMØs associated with antiviral host defense, including innate immune response, complement activation, inflammatory response, and chemotaxis. Antiviral host defense genes that were downregulated in AMØs from Zfhx3^+/+^ mice, but not in Zfhx3^–/–^ AMØs, include Ripk3 (30), Msr1 (31), Cxcl17 (32), C1q (33), C4a (34), C4b (35), Cfh (36), Ch25h (37), Ccl17 (38), and Cd200 (39). A number of genes protected from hypercapnic suppression in AMØs from Zfhx3^–/–^ mice also contribute to resolution of inflammatory injury associated with lung infections by promoting apoptosis and clearance of apoptotic cells. These include Ripk3 (45), Msr1 (46, 47), Cd36 (48), and Ptafr (48).

In addition to the gene regulation in AMØs demonstrated by RNA-Seq, we showed that hypercapnia inhibited expression of Ifnb1 and Rsad2 (49, 50) mRNA and Oas1 (49, 50) protein in the lungs of Zfhx3^+/+^ mice infected with IAV and that hypercapnic suppression of these antiviral mediators was blocked in IAV-infected myeloid Zfhx3^–/–^ mice. Correspondingly, hypercapnia increased IAV replication and expression of viral ns1 RNA and NS1 and M2 protein in the lungs of IAV-infected Zfhx3^+/+^ mice, while Zfhx3^–/–^ mice were protected against the CO_2_-induced increases in viral replication and IAV gene and protein expression. Moreover, Zfhx3^–/–^ mice breathing 10% CO_2_ exhibited less severe lung injury and lower mortality than CO_2_-breathing Zfhx3^+/+^ mice infected with IAV. The fact that LysM-Cre–mediated deletion of Zfhx3 attenuated the hypercapnia-induced increase in lung injury and mortality of IAV infection underscores the importance of myeloid cells as a key target of elevated CO_2_ in suppressing antiviral host defense. Indeed, a critical role of AMØs in host defense against influenza viruses has been established by studies in several species showing that ablation of AMØs with intratracheal clodronate or by genetic strategies increases the mortality of IAV infection (25–28). The protective effects of AMØs involve both robust type I IFN antiviral signaling and modulation of the inflammatory response to viral infection. While IAV can infect mouse and human AMØs, most studies show that infection is largely abortive, resulting in release of minimal amounts of infectious new virions (51). However, elevated CO_2_ suppresses the AMØ antiviral response, such that BAL AMØs obtained 1 day after intratracheal inoculation of hypercapnic mice with IAV expressed increased amounts of viral NS1 and M2 and released 6-fold more viable virus than AMØs from mice breathing ambient air (Figure 5, D and E, and Supplemental Figure 7). The increase in mRNA and protein expression of NS1, a nonstructural viral gene product that inhibits transcription of antiviral host genes and blocks the activity of IFN-stimulated antiviral effectors (52), is indicative of active viral replication in AMØs from hypercapnic mice. Importantly, myeloid Zfhx3 deficiency prevented hypercapnia from suppressing antiviral gene and protein expression, and from increasing viral NS1 and M2 expression and viral replication in AMØs from IAV-infected mice. Taken together, these findings indicate that AMØs are a key cellular target of CO_2_ in inhibiting host defense against IAV and that deletion of Zfhx3 abrogates suppression of the antiviral response in this myeloid cell population. Neutrophils infiltrate the lung following influenza virus infection (53) and to contribute to influenza-induced lung injury in humans and mice (54–56). Importantly, we found that Zfhx3 is expressed in mouse neutrophils and that LysM-Cre–mediated deletion ablates neutrophil expression of the gene. However, the importance of neutrophils as a target of CO_2_ in the context of viral infection is uncertain, since they are not known to be infected by IAV and evidence that they play a role in host defense against the virus is conflicting (57, 58). Of note, we were unable to recover sufficient numbers of intact neutrophils from the lungs of IAV-infected mice to determine the effect of Zfhx3 deficiency on antiviral gene expression or the presence of viable IAV in these cells.

Because LysM is expressed in a fraction of AT2 cells, LysM-Cre–mediated recombination could have deleted Zfhx3 in this AT2 cell subset, such that this might contribute to protection from the adverse effects of hypercapnia in Zfhx3^–/–^ mice. However, Zfhx3 protein expression was not decreased in AT2 cells of Zfhx3^–/–^ mice as compared with Zfhx3^+/+^ mice, in contrast to the > 95% reduction in Zfhx3 mRNA and protein in Zfhx3^–/–^ MØs and neutrophils. Furthermore, only a handful of genes were differentially expressed in AT2 cells from myeloid Zfhx3^–/–^ as compared with Zfhx3^+/+^ mice (Supplemental Figure 2C), and exposure of Zfhx3^+/+^ mice to normoxic hypercapnia for 7 days had no effect on gene expression in AT2 cells (Supplemental Figure 2D), in marked contrast to AMØs. This suggests that the decreases in viral protein expression observed in lung epithelial cells of myeloid Zfhx3^–/–^ mice exposed to CO_2_ and infected with IAV (Figure 5B and Supplemental Figure 6, B and D) may have resulted from an indirect effect of intact rather than suppressed antiviral activity in Zfhx3-deficient MØs (and possibly other myeloid cells). The foregoing results are also consistent with the conclusion that selective deficiency of Zfhx3 in the myeloid lineage is sufficient to reduce IAV-induced lung injury and mortality in the setting of hypercapnia.

Beyond MØs and AT2 cells, single-cell RNA-Seq confirms that Zfhx3 is expressed in a broad array of cell types, including ciliated and mucus-secreting airway epithelial cells, fibroblasts, smooth muscle cells, adventitial cells, and others, during human lung development (59) and in adult mouse and human lungs (60, 61). Widespread expression among many cell types during development and in the adult lung suggests that Zfhx3 has multiple functions, as would be expected for such a large and complex protein. Furthermore, Zfhx3 may mediate CO_2_-induced changes in gene expression in lung cells other than AMØs that also affect antiviral host defense. Indeed, we previously showed that hypercapnia alters global gene expression in human bronchial epithelial cells, including downregulation of innate immune pathways (62), and increases viral uptake in lung epithelial cells (63). In addition, while exposure of mice to 10% CO_2_ for 7 days had no effect on AT2 cell gene expression in the current study, we recently reported that exposure to hypercapnia for 21 days altered expression of numerous genes in AT2 cells (44). Thus, future studies should examine the role of Zfhx3 as a mediator of responses to elevated CO_2_ in airway and alveolar epithelial cells, as well as other cell types, in the context of viral infection.

We chose to investigate the role of Zfhx3 as a potential mediator of CO_2_ effects on the immune system in mice because of its homology to *Drosophila* zfh2, which we previously identified as a mediator of CO_2_-induced suppression of host defense against bacterial infection in the fly (19). While full-length human Zfhx3 (Zfhx3-A) has 23 zinc fingers and 4 homeodomains, as compared with 16 zinc fingers and 3 homeodomains in zfh2 (64), key regions of the 2 proteins are highly homologous: homeodomains I, II, and III of Zfhx3 are, respectively, 77%, 69%, and 61% identical to the corresponding homeodomains in zfh2; 13 of the zinc fingers in Zfhx3 are 22%–89% identical to those in zfh2; and all of these domains in the 2 proteins are colinearly arranged (20). Knockdown of zfh2 abrogated CO_2_-induced suppression of multiple antimicrobial peptides in cultured *Drosophila* S2 phagocytes (19), like the effect of Zfhx3 deletion on hypercapnic suppression of innate immune and antiviral genes in mouse and human MØs. Furthermore, in vivo knockdown of zfh2 in the immune responsive fat body protected *Drosophila* from the increase in mortality of bacterial infection caused by elevated CO_2_ (19), similar to the effect of myeloid Zfhx3 deficiency on the hypercapnia-induced increase in mortality of IAV infection in mice. The high degree of homology between zfh2 and Zfhx3 and the parallels between the effect of knocking down or knocking out the respective genes in *Drosophila* and mice strongly suggest that the role of Zfhx3 as a mediator of CO_2_ effects on the immune system has been evolutionarily conserved.

Its large size and multiplicity of homeodomains and zinc fingers suggests the possibility of numerous protein-DNA and protein-protein interactions through which Zfhx3 may mediate CO_2_ effects on gene and protein expression. Several different Zfhx3-DNA and -protein interactions have been elucidated in previous studies unrelated to CO_2_ or the immune system. ATBF1, the alternate name for Zfhx3, derives from the observation that the protein negatively regulates transcription of α-fetoprotein (AFP) by binding to AT-rich elements in the AFP promoter and enhancer (65). Alternately, ATBF1 was shown to bind to a leucine zipper motif of the oncoprotein v-Myb and thereby inhibit its oncogenic transcriptional activity (66). Also, ATBF1 was found to bind to and inhibit transcriptional activity of the estrogen receptor by blocking its ability to interact with the steroid receptor coactivator AIB1 in breast cancer (67). Another study showed that, upon stimulation with TGF-β, ATBF1 bound the transcription factor RUNX3, resulting in cotranslocation to the nucleus and synergistic upregulation of p21^Waf1/Cip1^ promoter activity in gastric cancer cells (68). These examples highlight the possibility that Zfhx3 may mediate effects of hypercapnia on transcription of immune and antiviral gene by direct binding to their promoters and/or other regulatory DNA elements or by interacting with other transcription factors or upstream components of a putative CO_2_-triggered signaling pathway.

The molecular mechanism by which elevated CO_2_ affects Zfhx3 remains to be elucidated. Notably, this mechanism is unlikely to be an effect of altered pH for 2 reasons. First, in previous studies with cultured cells, we have documented that hypercapnia-induced changes in gene expression are not a function of extracellular or intracellular acidosis (14, 16, 18). Second, in the current investigation, as in our previous in vivo studies (17, 18), mice were exposed to 10% CO_2_ for 3 days, allowing for maximal renal compensation of respiratory acidosis and return of pH to near normal, prior to IAV infection. One possible mechanism by which molecular CO_2_ might trigger alterations in gene expression is by carbamylation of regulatory proteins. Carbamylation by reaction of CO_2_ with the amino group of lysine residues represents a recently described posttranslational modification that can alter protein function (69, 70). Whether Zfhx3 or upstream components of a putative CO_2_ signaling pathway undergo differential carbamylation at normal versus elevated levels of PCO_2_ is another direction for future investigation.

In summary, in the present work, we show that KO of Zfhx3 in the myeloid lineage blocks hypercapnia-induced downregulation of antiviral and innate immune gene expression and protects mice from CO_2_-induced increases in viral replication, inflammatory lung injury, and mortality when infected with IAV. To our knowledge, these results establish Zfhx3 as the first known mediator of CO_2_ effects on the immune system in mammals and suggest that this function has been conserved across evolution from zfh2 in *Drosophila*. Further studies are needed to define additional components of the pathways through which elevated CO_2_ signals, including the CO_2_ sensor and the mechanism by which CO_2_ is sensed. This avenue of investigation has the potential to identify new targets for pharmacologic intervention to mitigate hypercapnia-induced immunosuppression, with the goal of improving outcomes of infection in patients with severe acute and chronic lung diseases.

## Methods

### Reagents.

All reagents are from Sigma-Aldrich, except where noted otherwise.

### Mice.

Zfhx3^fl/fl^ LyzM^Cre^ mice lacking Zfhx3 in the myeloid lineage (Zfhx3^–/–^ mice) were generated by crossing C57BL/6 Zfhx3^fl/fl^ mice (24) with LyzM^Cre^ mice (B6.129P2-Lyz2^tm1[cre]Ifo^/J, The Jackson Laboratory). Myeloid Zfhx3^–/–^ mice survive, grow, and appear phenotypically normal up to > 1 year of age. In all experiments, Zfhx3^fl/fl^ mice (Zfhx3^+/+^ mice) were used as controls. Of note, Zfhx3^+/+^ mice have the same response to hypercapnia and IAV infection as WT C57BL/6 mice, as described in our previous publication (18). Given that we observed no sex-related differences in responses to hypercapnia or outcomes of IAV infection in previous work with WT mice and in preliminary experiments with Zfhx3^–/–^ mice, mixed groups of male and female animals were used in all experiments. Mice were 6–12 weeks of age at the time of experimentation.

### Murine CO_2_ exposure.

Zfhx3^+/+^ and Zfhx3^–/–^ mice were exposed to normoxic hypercapnia (10% CO_2_/ 21% O_2_/ 69% N_2_) in a BioSpherix A environmental chamber (BioSpherix). O_2_ and CO_2_ concentrations in the chamber were maintained at the indicated levels using ProOx C21 O_2_ and CO_2_ controllers (BioSpherix). Age-and genotype-matched mice, simultaneously maintained in air, served as controls in all experiments.

### In vivo influenza virus infection.

Mice preexposed to ambient air or hypercapnia for 3 days were anesthetized with isoflurane, intubated with a 20-gauge Angiocath catheter, and inoculated intratracheally with mouse-adapted IAV strain A/WSN/33 (H1N1; gift of Robert Lamb; Northwestern University, Evanston, Illinois, USA) or PBS as control, before being maintained under air or hypercapnia exposure conditions, as previously described (18). Depending on the experiment, mice were infected with either 3, 30, or 300 pfu/animal in 50 μL of PBS.

### Clinical assessment of IAV infection.

After infection with IAV, mice were weighed daily and monitored every 8 hours for signs of severe distress (slowed respiration, failure to respond to cage tapping, failure of grooming, and/or fur ruffling). Mice that developed severe distress were considered moribund and sacrificed, and the deaths were recorded as IAV-induced mortality. Mice that died between monitoring episodes were also recorded as IAV-induced mortality.

### Cells.

AMØs were isolated by BAL in mice anesthetized with ketamine and xylazine. Tracheotomy was performed, and a 26-gauge catheter was inserted into the trachea and secured with vinyl suture. Ice-cold PBS (1 mL) was instilled and withdrawn serially 3 times. BAL fluid was centrifuged at 350 g for 10 min and 4°C, and cells were allowed to adhere to plastic, nonadherent cells were removed, resulting in an adherent AMØs population ≥ 98% pure. AMØs were cultured in RPMI 1640 (ATCC) with 10% FBS, 2 mM L-glutamine, 1 mM sodium pyruvate, 20 μM 2-ME, 100 U/mL penicillin, and 100 μg/mL streptomycin (RPMI media) (14). Cells were rested for 24 hours to allow the transient proinflammatory profile of freshly isolated AMØs to subside prior experimentation (71). BAL cells were also centrifuged in a cytospin (350 g for 5 minutes at 4°C) and fixed in 4% PFA. For mouse BMDM, BM was isolated from Zfhx3^+/+^ or Zfhx3^–/–^ mice following a standard protocol (72). Cells were plated in 10 cm tissue culture plates at a density of 3 × 10^6^ cells/ plate. Cells were cultured with L929 cell supernatants/RPMI/FBS media to induce MØ differentiation. Media were changed every 3 days, and BMDM were harvested by scraping on day 6, followed by plating in 24-well plates at a density of 8 × 10^5^ cells/well.

Human AMØs were obtained by BAL from the contralateral lung of patients undergoing bronchoscopy for clinical diagnosis of noninfectious focal lung lesions (14, 73) under a protocol approved by the Northwestern University IRB and cultured as for mouse AMØs. Human monocytic leukemia THP-1 cells (ATCC) were cultured in RPMI media and differentiated to a MØ phenotype by exposure to 5 nM PMA for 48 hours (14).

### Exposure of cells to normocapnia and hypercapnia.

Normocapnia consisted of standard incubator atmosphere: humidified 5% CO_2_ (PCO_2_ 36 mmHg)/95% air, at 37°C. Hypercapnia consisted of 15% CO_2_ (PCO_2_ 108 mmHg)/21% O_2_/64% N_2_. Cells were exposed to hypercapnia in an environmental chamber (C-174, BioSpherix) contained within the same incubator where control cultures were simultaneously exposed to normocapnia. In all cases, cells were exposed to hypercapnia or maintained in normocapnia as control for 18 hours prior to infection with IAV. All media were presaturated with 5% or 15% CO_2_ before addition to the cells.

### In vitro influenza virus infection.

MØs were infected with IAV A/WSN/1933 (H1N1) at 2 MOI per cell, using a single-cycle protocol. Cells were incubated with IAV in a 5 % or 15% CO_2_ atmosphere at 37°C for 1 hour, during which time plates were rocked every 15 minutes to distribute the virus evenly and to keep the monolayer moist. Cells were then washed twice with PBS to remove excess of virus, and fresh RPMI media was added to the plates.

### Immunofluorescence microscopy in tissue sections and cell cultures.

Sections were deparaffinized with xylene, rehydrated by using graded ethanol, and subjected to heat-induced antigen retrieval with 10 mM of sodium citrate buffer (pH 6.0). After blocking with BSA 1% (wt/vol) in PBS, tissues were incubated overnight with anti-Zfhx3, anti-NS1, or anti-M2 antibodies. Complete antibody information is provided in the Supplemental Table 1. Then, sections were washed with PBS, and Alexa Fluor–labeled secondary antibodies (1 μg/mL) were added. Colabeling with F4/80 or Chil3 (MØ markers) and SPC (AT2 cell marker) was achieved by the addition of anti-F4/80, anti-Chil3, or anti-SPC antibodies, respectively. After washing, Alexa Fluor–conjugated antibodies (1 μg/mL) were added, and sections were incubated for 1 hour at room temperature.

Cells were fixed with 4% PFA, permeabilized with 0.1% Triton X100 for 5 minutes, blocked with BSA 1% (wt/vol) in PBS, and incubated overnight with specific antibodies against host Zfhx3, Oas1, Rsad2, and Chil3 and viral NS1, M2, and NP. Then, sections were washed with PBS, and Alexa Fluor–labeled secondary antibodies were added (Supplemental Table 1). Nuclei were visualized with DAPI, and slides were mounted with Gel/Mount (Biomeda). Nonimmune mouse, rabbit, or goat IgGs were used as a negative control, and the staining was negative for all nonimmune controls, regardless of protocol. Fluorescence images were obtained using Axiovert 200M Fluorescence Microscope (Zeiss). Images were obtained using the same exposure time for all samples from a given experimental set. To avoid saturation, the exposure time was selected based on the most brightly stained sample and used for all other samples in the set. This approach results in equal subtraction of background autofluorescence from all images.

### Multiplexed in situ hybridization RNAscope Assay combined with immunofluorescence on tissue sections.

RNAscope Multiplex V2 Assay (Advanced Cell Diagnostics) was performed on paraffin-embedded 5 μm slices of lung tissue using mild digest times according to manufacturer instructions. Hybridization was detected with Opal dyes from Akoya Biosciences. The following RNAscope Probes were used: Mm-Rsad2 with Opal dye 520, Mm-Ifnb1-C2 with Opal dye 570, and V-Influenza-H1N1-NS2NS1-C3 with Opal dye 620. The RNA-Protein Codetection Ancillary kit was used to colabel RNA with viral M2 and mouse Chil3 proteins. Nuclei were stained with DAPI. Images were acquired using an Axiovert 200M Fluorescence Microscope (Zeiss) or a Nikon A1C Confocal Microscope at the Center for Advanced Microscopy at Northwestern University Feinberg School of Medicine. Final images were rendered using Fiji.

### Immunoblotting.

The presence of indicated proteins in lung or cell homogenates were assessed by immunoblotting using the following antibodies: anti-Zfhx3, anti–β-actin, and anti-Oas1. Complete antibody information is provided in Supplemental Table 1. Membranes were incubated with IRDye (1:10,000, LI-COR) Biosciences or HRP-conjugated (1:5,000) secondary Abs for 1 hour at room temperature. Signals were captured using the LI-COR Odyssey Fc Imager and analyzed with ImageStudio software (LI-COR).

### qPCR.

RNA was extracted using RNeasy Mini Kit (Qiagen) and reverse transcribed using an iScript cDNA Synthesis Kit (Bio-Rad). Amplification was performed using the CFX Connect Real-Time System (Bio-Rad) and TaqMan FAM-labeled primer/probes sets Mm01240016_m1 for Zfhx3 and Mm00607939_s1 for β-actin. Zfhx3 gene expression was normalized to β-actin. Relative expression was calculated by the ΔΔCT method (74).

### Alveolar MØ isolation by flow cytometry.

Tissue preparation for flow cytometry analysis and cell sorting was performed as previously described (75). Cells were stained with eFluor 506 (eBioscience) viability dyes, incubated with FcBlock (BD Biosciences), and stained with fluorochrome-conjugated antibodies as described in detail previously (75). Data were acquired on a BD LSR II flow cytometer (for information regarding instrument configuration and antibody panels, see ref. 75). Compensation, analysis, and visualization of the flow cytometry data were performed using FlowJo software (BD Biosciences). Fluorescence minus one controls were used when necessary to set up gates. Cell sorting was performed at Northwestern University RLHCCC Flow Cytometry core facility on SORP FACSAria III instrument (BD Biosciences) with the same optical configuration as for flow cytometry on the LSR II, using a 100 μm nozzle and a pressure of 40 psi.

### AT2 cells isolation by flow cytometry.

Tissue preparation for mouse AT2 isolation was performed as described previously (44). Briefly, perfused lungs were treated with 50 U/mL dispase (Corning, 47743-724) and 0.25 mg/mL DNase (D4513-1VL) before being manually dissected and minced. Single-cell suspensions were enriched for epithelial cells using anti-EpCAM magnetic microbeads (Miltenyi Biotec, 130-105-958). Cell sorting was performed with a BD FACSAria cell sorter using BD FACSDIVA software (BD Biosciences).

### Neutrophil isolation by MACS separation.

BM was obtained from Zfhx3^+/+^ or Zfhx3^–/–^ mice following a standard protocol (72). Neutrophils were isolated by depletion of nontarget cells using the Neutrophil Isolation Kit (Miltenyi Biotec) following the manufacturer’s protocol, in which magnetically labeled nontarget cells are retained within a MACS Column in the magnetic field of a MACS Separator, while unlabeled neutrophils run through the column.

### RNA-Seq library preparation.

RNA quality and quantity were measured using High Sensitivity RNA ScreenTape and the Agilent 4200 Tapestation System (Agilent Technologies). Briefly, mRNA was isolated from 50 ng of purified total RNA using oligo-dT beads (New England Biolabs). NEBNext Ultra RNA kit was used for full-length cDNA synthesis and library preparation. Libraries were pooled, denatured, and diluted, resulting in a 2.0 pM DNA solution. PhiX control was spiked at 1%. Libraries were sequenced on an Illumina NextSeq 500 instrument (Illumina) using NextSeq 500 High Output reagent kit 75 cycles (Illumina) with a target read depth of approximately 5–10 million aligned reads per sample.

### Gene expression profiling (RNA-Seq) and bioinformatic analysis.

Computation intensive analysis was performed using “Genomics Nodes” on Northwestern’s High Performance Computing Cluster, Quest (Northwestern IT and Research Computing). Reads were demultiplexed using bcl2fastq, quality was assessed using FastQC, reads were trimmed and aligned to mm10 reference genome using TopHat2, read counts were associated with genes using the GenomicRanges (76), and differential gene expression was assessed using edgeR (77, 78) R/Bioconductor packages. Genes with less than 1 normalized read count across at least half of the samples were filtered from all analyses. Pearson correlation matrix and clustering heatmaps were built using Morpheus software (https://software.broadinstitute.org/morpheus). Overrepresentation analysis (ORA) of GO terms from biological processes of all genes downregulated or upregulated by hypercapnia were separately analyzed using the Gene Ontology Analysis InnateDB tool (79), which utilizes a manually curated knowledgebase of genes, proteins, interactions, and signaling pathways involved in mammalian innate immune responses. Results from the InnateDB analysis were confirmed using GeneGo Metacore (Clarivate), a separately curated database and pathway analysis tool. RNA-Seq data have been deposited to the NCBI Gene Expression Omnibus (GEO) complied with MIAME standards (accession nos. GSE183922 for AMØs and GSE243969 for AT2 cells). From the GO biological term results, 5–6 processes of interest were selected. Interaction networks were constructed for each set of DEGs associated with these 5–6 terms using the GeneMANIA plug-in (80) for Cytoscape 3.8.0 software (81). GeneMANIA can find other genes that are related to the set of input genes and produce a functional association network based on their relationships, such as pathways, coexpression, colocalization, genetic interaction, physical interaction, and shared protein domains, based on the published literature.

### Lung histopathology.

Mice were euthanized, and lungs were perfused via the right ventricle with 10 mL HBSS with Ca^2+^ and Mg^2+^. A 20-gauge angiocatheter was inserted into the trachea and secured with a suture, the heart and lungs were removed en bloc, and lungs were inflated with up to 0.8 mL of formalin at a pressure not exceeding 16 cm H_2_O and fixed overnight at 4°C. Tissues were embedded in paraffin, sectioned (4 μm thickness), deparaffinized, and stained with H&E by the Northwestern University Mouse Histology and Phenotyping Laboratory. Images of lungs were obtained using the TissueFAXS PLUS Scanning System (TissueGnostics) at the Northwestern University Center for Advanced Microscopy. Serial images (~50 per whole lung section) were stitched into a high-resolution macroscopic montage. The severity of inflammatory lung injury was evaluated by observers blinded to experimental groups using a validated HPS in which each of the ~50 images in a representative section is assigned a score of 0–26 (least to most severe) based on assessment of quantity and quality of peribronchial inflammatory infiltrates, luminal exudates, perivascular and parenchymal infiltrates, and thickening of the membrane wall, as described previously (82, 83). The scores for all images of each lung section were then averaged, and the composite score for that animal’s lung was considered as 1 data point for statistical analysis and an individual data point in HPS graphs. This scoring system has been previously validated for assessment of murine lung injury caused by IAV and other respiratory pathogens (18, 83, 84).

### Preparation of lung homogenates for viral plaque assay and Western blot.

Mice were euthanized, the inferior vena cava was incised, and the right ventricle was perfused in situ with > 1 mL of sterile PBS. Lungs were removed and kept on ice prior to and during homogenization for 30 seconds in PBS. The homogenate was split into 2 aliquots, and an additional 1 mL of PBS was added to one of the aliquots, which was then centrifuged (450 g for 10 minutes, at 4°C). Madin-Darby canine kidney (MDCK) cells were grown in 6-well plates to 100% confluence and then incubated with serial 10-fold dilutions of lung homogenate in DMEM and 1% bovine serum albumin (BSA) for 1 hour (37°C). Supernatants were then aspirated, the cells were washed with PBS, 3 mL of replacement media (2.4% Avicel [IMCD], 2× DMEM, and 1.5 μg of N-acetyl trypsin) were added to each well, and the plates were incubated for 3 days. The overlay was then removed, and viral plaques were visualized using naphthalene black dye solution (0.1% naphthalene black, 6% glacial acetic acid, 1.36% anhydrous sodium acetate) (85). The second aliquot of lung homogenate was mixed with 0.5 mL RIPA buffer and used for immunoblotting.

### Statistics.

Statistical analyses were carried out using Prism software (GraphPad Prism 9.0). Data are presented as mean ± SD. Differences between 2 groups were assessed using a 2-tailed Student’s *t* test. Levene’s test was used to analyze the homogeneity of variances. Differences between multiple groups were assessed by 1-way ANOVA followed by Sidak’s multiple-comparison test. Survival was analyzed using log-rank test. Significance was accepted at *P* < 0.05.

### Study approval.

The work was performed according to a protocol approved by the IACUC of Northwestern University and is compliant with the *Guide for the Care and Use of Laboratory Animals* (National Academies Press, 2011). Human AMØs were obtained by BAL from the contralateral lung of subjects undergoing bronchoscopy for clinical diagnosis of noninfectious focal lung lesions under a protocol approved by the Northwestern University Institutional Review Board (Chicago, Illinois).

### Data availability

Values for all data points in graphs are reported in the Supporting Data Values file.

## Author contributions

SMCM and PHSS conceived and designed the research. SMCM, FC, FJGG, AN, and HAV performed the experiments. SMCM and HM performed the bioinformatics analysis. SMCM and PHSS analyzed and interpreted the data. GRSB and GJB contributed reagents or analytic tools. SMCM and PHSS wrote the manuscript. All authors provided edits and feedback on the manuscript.

## Supplementary Material

Supplemental data

Unedited blot and gel images

Supporting data values

## Version 1 01/16/2024

In-Press Preview

## Version 2 02/22/2024

Electronic publication
