# Supplementary material for: Myeloid Zfhx3 deficiency protects against hypercapnia-induced suppression of host defense against influenza A virus
Source: JCI Insight. 2024 Jan 16;9(4):e170316. doi: 10.1172/jci.insight.170316 (PMC11143927; doi:10.1172/jci.insight.170316)
Supplement: Supplemental data [file jciinsight-9-170316-s044.pdf]

Myeloid *Zfhx3* Deficiency Protects Against Hypercapnia-induced Suppression of Host Defense Against Influenza A Virus

S. Marina Casalino-Matsuda, Fei Chen, Francisco J. Gonzalez-Gonzalez, Hiroaki Matsuda, Aisha Nair, Hiam Abdala-Valencia, G. R. Scott Budinger, Jin-Tang Dong, Greg J. Beitel and Peter H. S. Sporn

Supplemental Figures and Table:

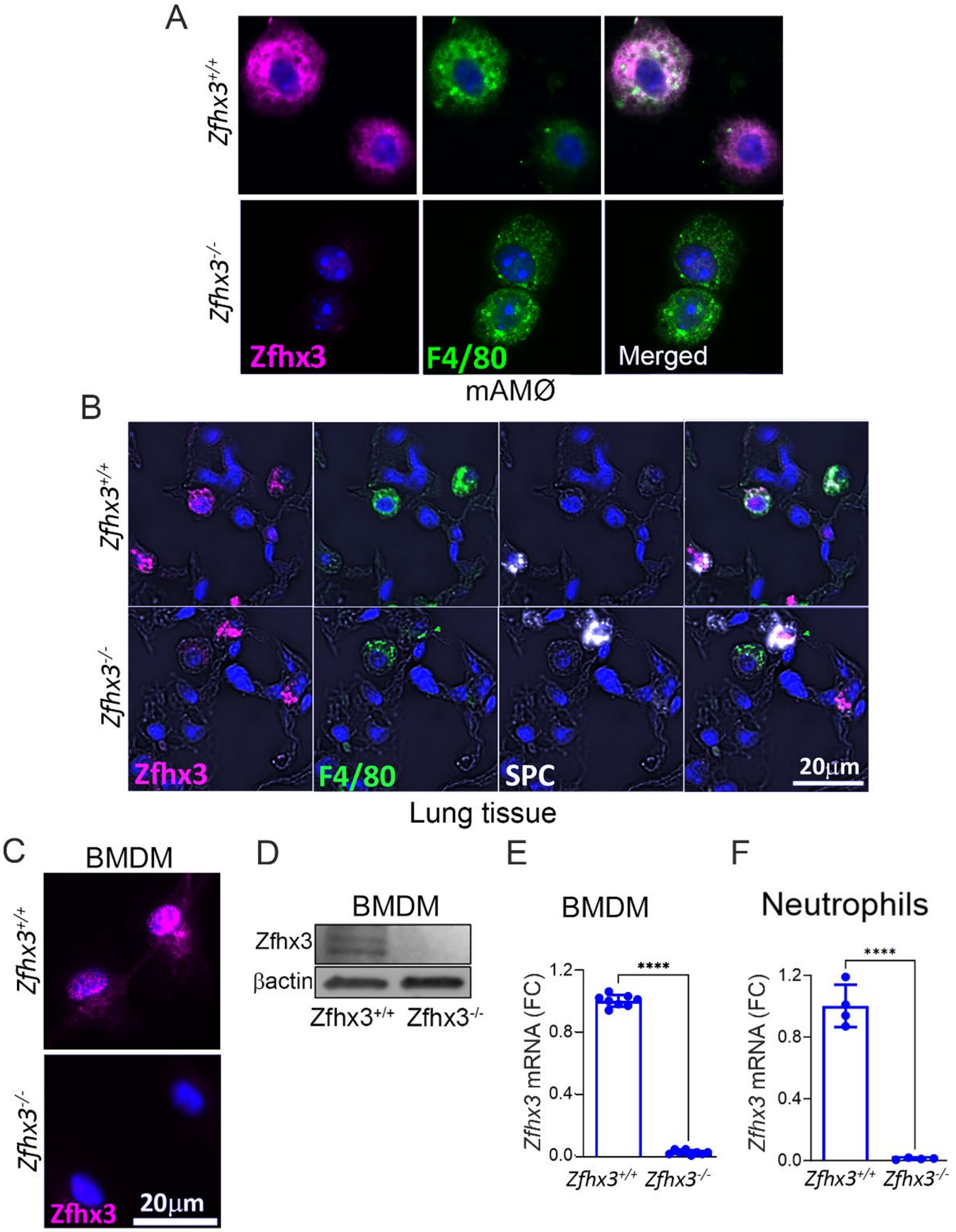

<u>Supplemental Figure 1:</u> ***Zfhx3* protein and mRNA are markedly reduced in macrophages and neutrophils from *Zfhx3$^{fl/fl}$LyzM^{Cre}* (*Zfhx3$^{-/-}$*) mice.** BAL AMØs, lung tissue, BMDM and bone marrow neutrophils were obtained from *Zfhx3$^{+/+}$* and *Zfhx3$^{-/-}$* mice. AMΦs (A), lung tissue sections (B) and BMDM (C) stained for Zfhx3 (magenta), F4/80 (green, MØs) and SPC (white, AT2 cells). Nuclei stained with DAPI (blue). Zfhx3 protein (D) and *Zfhx3* mRNA (E) expression in BMDMs measured by immunoblot and qPCR respectively. Zfhx3 expression was also measured in neutrophils by qPCR (F). Student's t test (two tailed), ****P<0.0001. FC=fold change.

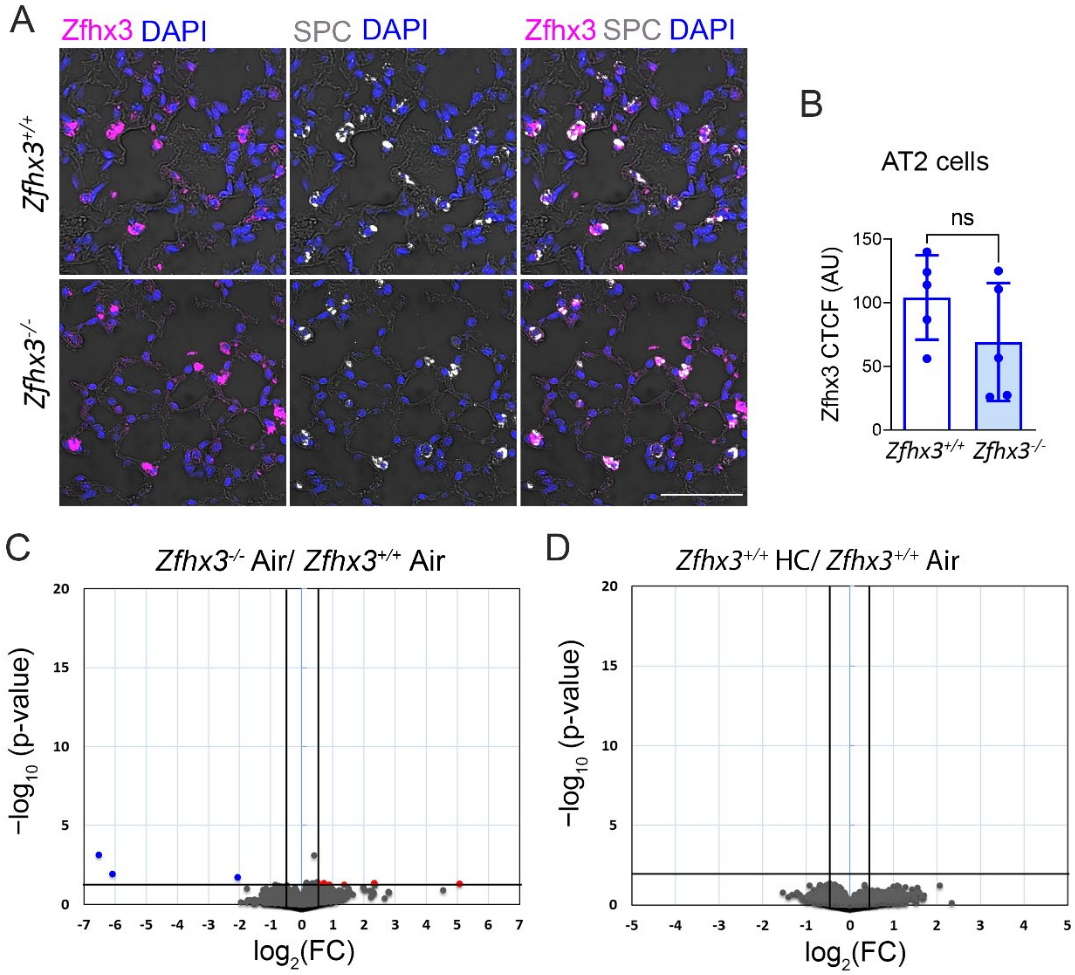

<u>Supplemental Figure 2:</u> **Myeloid-targeted-*Zfhx3* deficiency in *Zfhx3$^{fl/fl}$LyzM^{Cre}* (*Zfhx3$^{-/-}$*) mice does not decrease expression of Zfhx3 protein or mRNA and minimally alters global gene expression in AT2 cells.** Lung tissue was obtained and AT2 cells were isolated by flow cytometry from *Zfhx3$^{+/+}$* and *Zfhx3$^{-/-}$* mice. Lung tissue sections were immunostained for Zfhx3 (magenta) and SPC (white, AT2 cells) and nuclei were labeled with DAPI (blue), scale bar = 50 µm. (A). AT2 cells from Zfhx3$^{+/+}$ and Zfhx3$^{-/-}$ mice were immunostained for Zfhx3, and Zfhx3 protein was quantified as corrected total cell fluorescence (CTCF) expressed in arbitrary units (AU) from 200 cells from 5 independent experiments (B) and analyzed by two tailed Student's test. AT2 cells from *Zfhx3$^{+/+}$* and *Zfhx3$^{-/-}$* mice exposed to ambient air or normoxic hypercapnia (10% CO_2/ 21% O_2, HC) for 7 days were subjected to RNA sequencing. Volcano plots showing statistical significance (−log10 [P value]) plotted against log2 fold change of gene expression for AT2 cells from *Zfhx3$^{-/-}$* vs *Zfhx3$^{+/+}$* mice breathing ambient air (C) and AT2 cells from *Zfhx3$^{+/+}$* mice exposed to hypercapnia vs air (D). In panel C, upregulated genes (log2 [fold change] ≥+0.5, adjusted P value <0.05) are shown in red and downregulated genes (log2 [fold change] ≤−0.5, adjusted P value <0.05) are shown in blue.

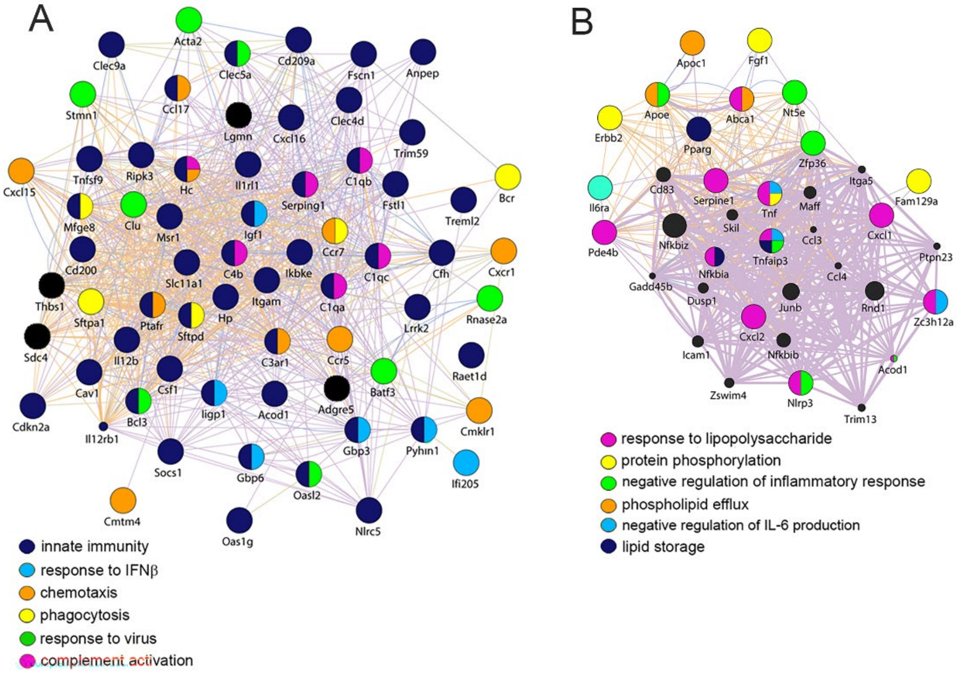

<u>Supplemental Figure 3</u>: **Gene networks downregulated and upregulated in alveolar macrophages from mice exposed to 10% CO_2 for 7 days.** AMØs were isolated by flow cytometry from lungs of *Zfhx3*$^{+/+}$ mice exposed to ambient air or normoxic hypercapnia (10% CO_2/ 21% O_2, HC) for 7 days and subjected to RNA sequencing. Gene networks of GO biological processes downregulated (A) or upregulated (B) by hypercapnia.

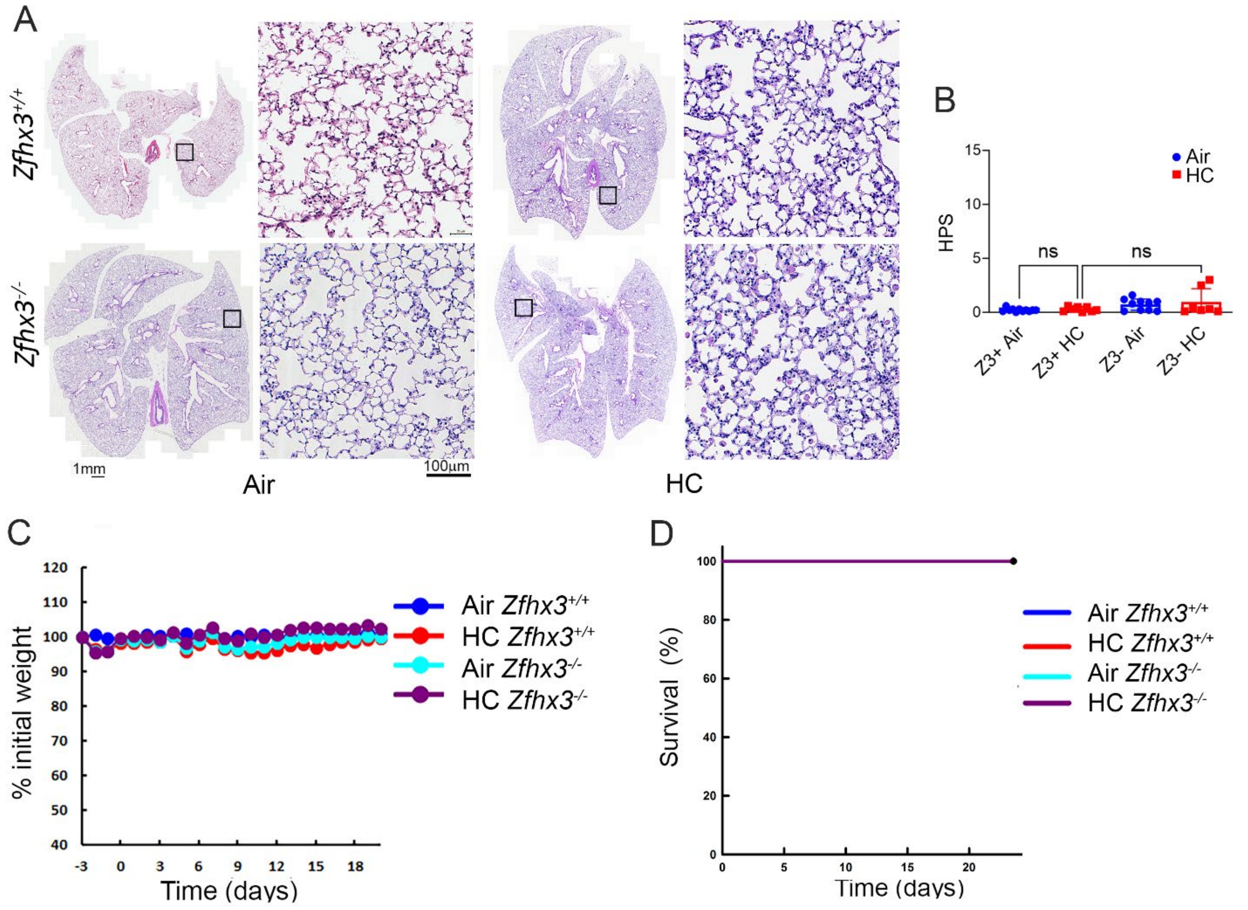

<u>Supplemental Figure 4:</u> Myeloid Zfhx3-deficient adult mice tolerate exposure to normoxic hypercapnia without illness or lung injury. *Zfhx3*$^{+/+}$ and *Zfhx3*$^{-/-}$ mice were exposed to ambient air or 10% CO_2 (hypercapnia, HC) for 7 days. Lungs were harvested 7 dpi, sectioned and stained with H&E; montage images of whole lung sections (A) were assessed blindly to determine histopathologic scores (HPS) for lung injury (B) analyzed by one way ANOVA plus Sidak's multiple comparisons test. Body weight changes over time did not differ between *Zfhx3*$^{+/+}$ and *Zfhx3*$^{-/-}$ mice exposed to air or hypercapnia (C), and Kaplan-Meier plot shows that all animals in all 4 groups remained alive at the end of the experiment (D).

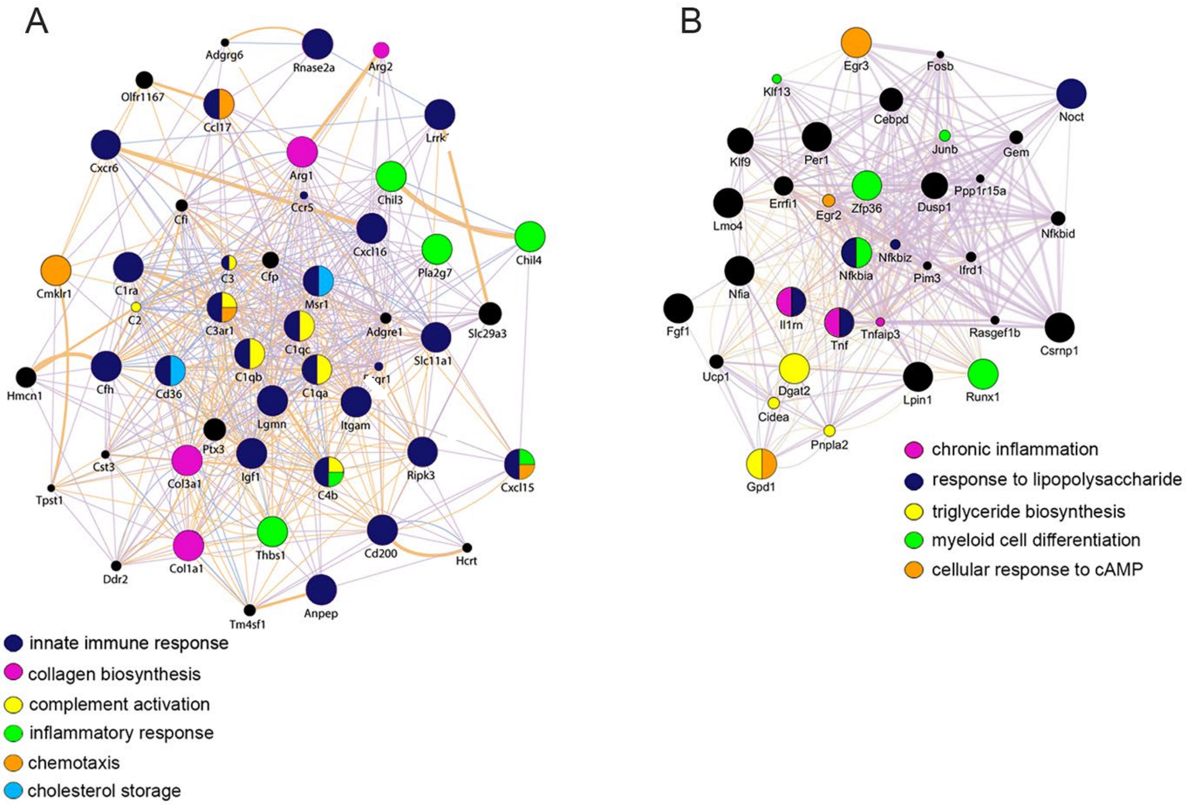

Supplemental Figure 5: **Gene networks for which myeloid *Zfhx3* deficiency abrogates hypercapnia-induced transcriptional alterations in alveolar macrophages.** AMØs were isolated by flow cytometry from the lung of *Zfhx3*$^{+/+}$ and *Zfhx3*$^{-/-}$ mice exposed to ambient air or normoxic hypercapnia (10% CO_2/ 21% O_2, HC) for 7 days and subjected to RNA sequencing. Gene networks of GO biological processes representing genes downregulated by hypercapnia in AMØs from *Zfhx3*$^{+/+}$ mice, but not those from *Zfhx3*$^{-/-}$ mice (A) and genes upregulated by hypercapnia in AMØs from *Zfhx3*$^{+/+}$ mice, but not those from *Zfhx3*$^{-/-}$ mice (B).

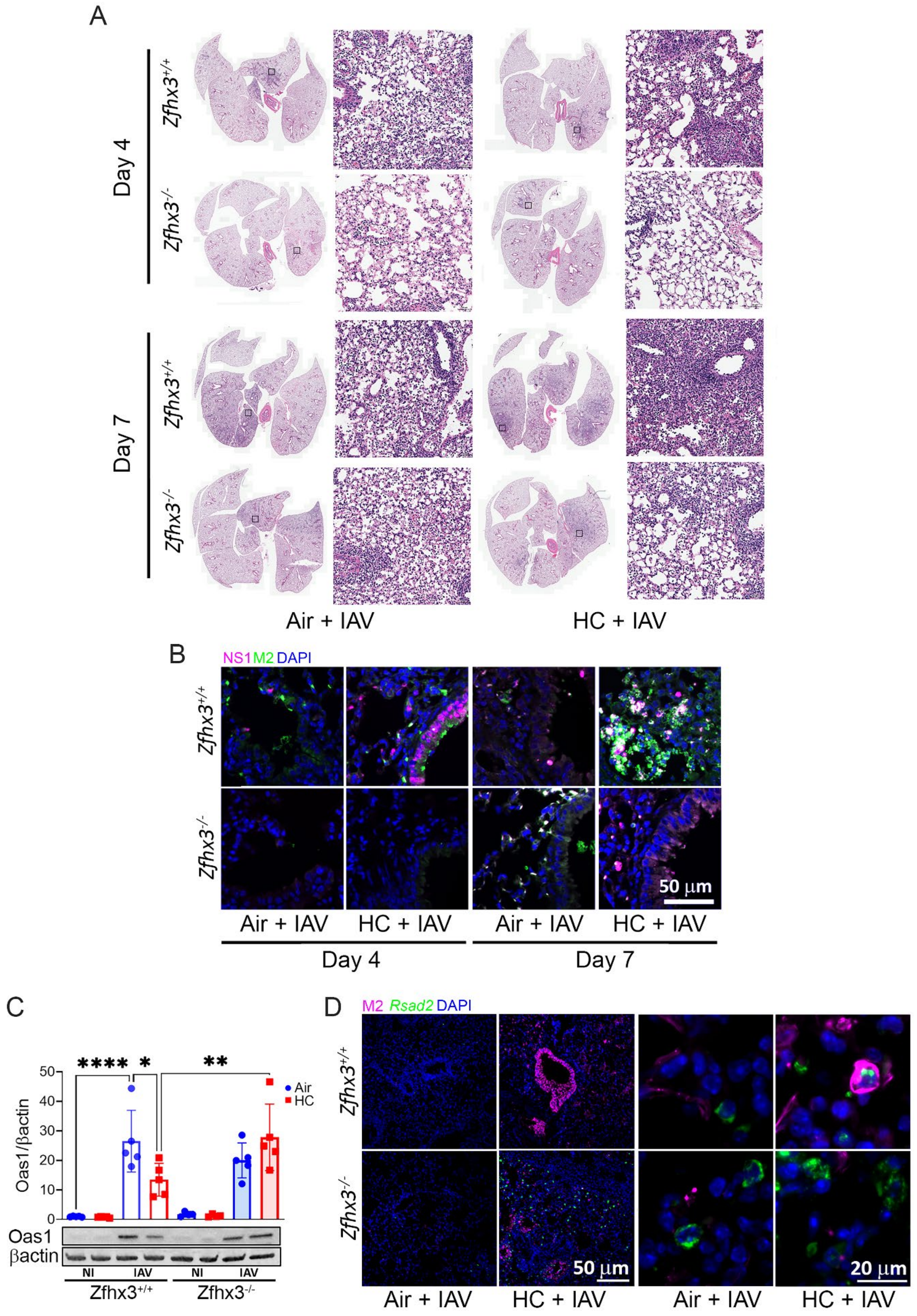
A
Day 4
Zfhx3+/+
Zfhx3-/-
Day 7
Zfhx3+/+
Zfhx3-/-
Air + IAV
HC + IAV
B
NS1 M2 DAPI
Zfhx3+/+
Zfhx3-/-
50 μm
Air + IAV
HC + IAV
Air + IAV
HC + IAV
Day 4
Day 7
C
**** * **
Air
HC
Oas1/βactin
50
40
30
20
10
0
Oas1
βactin
NI
IAV
NI
IAV
Zfhx3+/+
Zfhx3-/-
D
M2 Rsad2 DAPI
Zfhx3+/+
Zfhx3-/-
50 μm
20 μm
Air + IAV
HC + IAV
Air + IAV
HC + IAV

__Supplemental Figure 6__: **Myeloid *Zfhx3* deficiency protects against hypercapnia-induced suppression of antiviral gene and protein expression, and increases in viral protein expression and lung injury in IAV-infected mice.** *Zfhx3*$^{+/+}$ and *Zfhx3*$^{-/-}$ mice were pre-exposed to normoxic hypercapnia (10% CO_2/21% O_2, HC) for 3 days or ambient air as control, then infected intratracheally with 3 pfu IAV (A/WSN/33) per animal. Lungs from IAV-infected mice harvested 4 and 7 dpi were sectioned and stained with H&E; montage images of whole lung sections are shown (A). Expression of viral NS1 (magenta) and M2 (green) protein was assessed in lung tissue sections from mice sacrificed 4 and 7 dpi (B). Oas1 protein expression from homogenized lung tissue at 4 dpi was assessed by immunoblot and analyzed by one way ANOVA plus Sidak's multiple comparisons test; *P<0.05, ** P<0.01, **** P<0.0001 (C). Expression of *Rsad2* transcripts (green) and viral M2 (magenta) protein were assessed by RNAscope® and immunofluorescence microscopy respectively in lung tissue sections from mice sacrificed 4 dpi (D). Nuclei were stained with DAPI (blue).

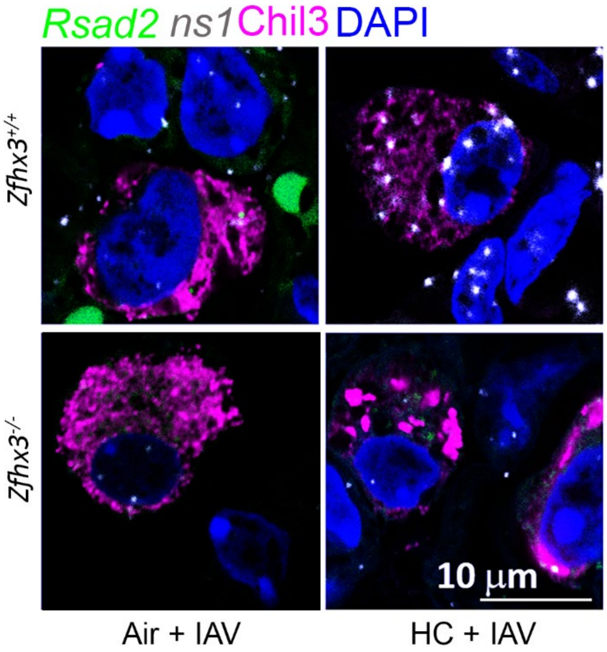

<u>Supplemental Figure 7</u>: Myeloid *Zfhx3* deficiency prevents hypercapnia-induced suppression of antiviral gene expression and increase of viral gene expression in IAV-infected mice. *Zfhx3*$^{+/+}$ and *Zfhx3*$^{-/-}$ mice were pre-exposed to normoxic hypercapnia (10% CO_2/21% O_2, HC) for 3 days, or ambient air as control, then infected intratracheally with 30 pfu of IAV (A/WSN/33) per animal; N = 6-10 per group. *Rsad2* (green) and viral *ns1* (white) transcripts and Chil3 protein (AMØ marker, magenta) were assessed by RNAscope® and immunofluorescence microscopy, respectively, in lung tissue sections from mice sacrificed 4 dpi. Nuclei were stained with DAPI (blue).

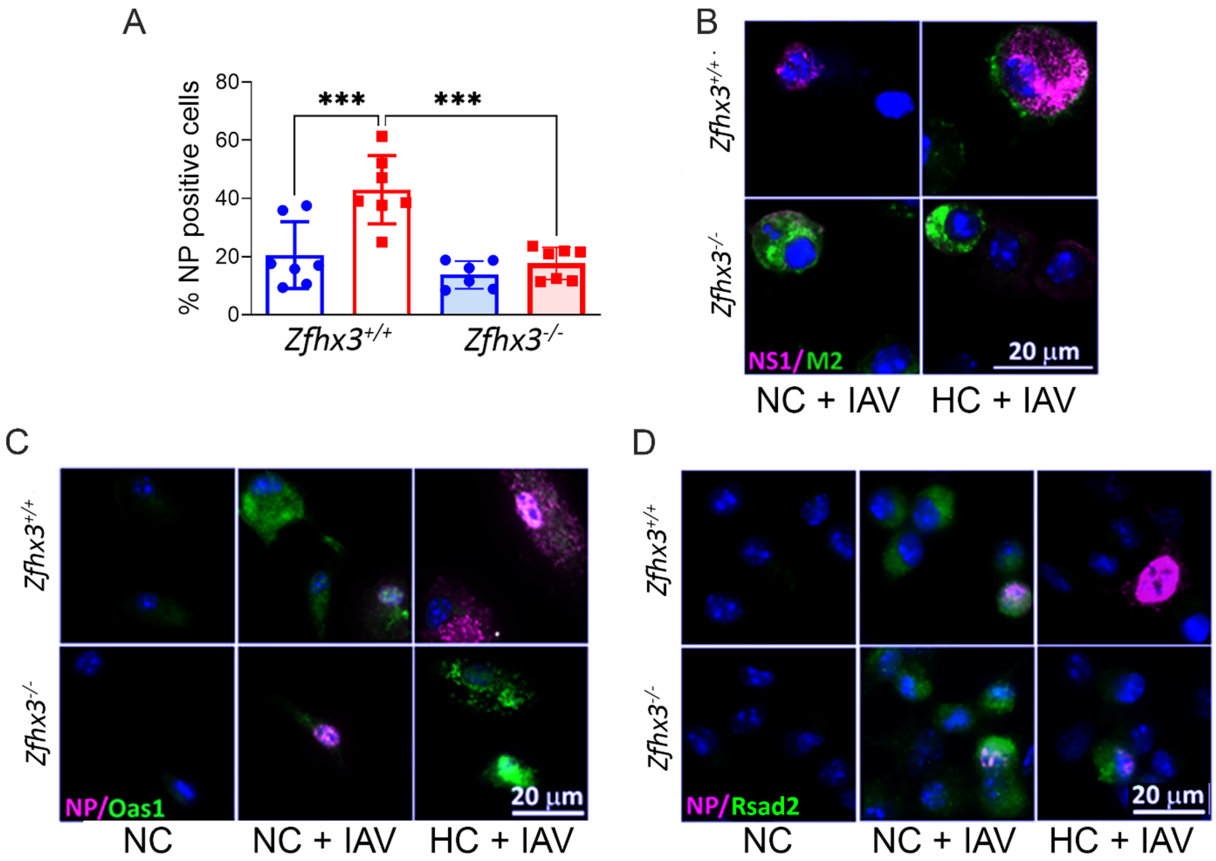

__Supplemental Figure 8:__ **Myeloid *Zfhx3* deficiency protects against hypercapnia-induced suppression of antiviral protein expression and increases in viral protein expression in IAV-infected mouse BMDM.** *Zfhx3*$^{+/+}$ and *Zfhx3*$^{-/-}$ BMDM were cultured under normocapnic (5% CO_2/95% air, NC) or hypercapnic (15% CO_2/21% O_2/64% N_2, HC) conditions for 18 h, infected with IAV, maintained in NC or HC for additional 24 h, then fixed and immunostained. NP expression was assessed by immunofluorescence microscopy, quantified using ImageJ, and analyzed by one way ANOVA plus Sidak's multiple comparisons test; *** $P<0.001$ (A). Representative images of BMDM immunostained for viral NS1 (magenta) and M2 (green) (B) and for NP (magenta), OAS1 (green) and Rsad2 (green) (C, D) are also shown.

Supplemental Table 1

**Antibodies**		
Rabbit polyclonal anti-NS1	GeneTex	Cat#GTX125990; RRID:AB_11170327
Mouse monoclonal anti-IAV M2	Invitrogen	Cat#MA1-082; RRID:AB_10892134
Mouse monoclonal anti-IAV NP	Abcam	Cat#ab128193; RRID:AB_11143769
Rat monoclonal anti-F4/80	Abcam	Cat#ab6640; RRID:AB_1140040
Goat polyclonal anti-SPC	Santa Cruz Biotechnology	Cat#Sc-7706; RRID:AB_2185507
Goat polyclonal anti-Chitinase 3-like 3/ECF-L	Novus	Cat#AF2446;<br>RRID: AB_2079008
Rabbit polyclonal anti-ATBF1 or ZFHX3 (D1-120)	MBL INTERNATIONAL	Cat#PD010;<br>RRID: AB_10598330
Rabbit polyclonal anti-ATBF1 or ZFHX3 (AT-6) Ab	MBL INTERNATIONAL	Cat#PD011; RRID:AB_10604524
Rabbit polyclonal anti-OAS1	Abcam	Cat#ab86343; RRID:AB_2158286
Rabbit polyclonal anti-Rsad2	Abcam	Cat#ab73864; RRID:AB_1640850
Goat anti-Rabbit IgG (H+L) Highly Cross-Adsorbed Secondary Antibody, Alexa Fluor Plus 555	ThermoFisher	Cat#A32732; RRID:AB_2633281
Donkey Anti-Mouse IgG (H+L) Antibody, Alexa Fluor 488 Conjugated	ThermoFisher	Cat#A21202; RRID:AB_141607
Rabbit anti-Goat IgG (H+L) Superclonal(TM) Secondary Antibody, Alexa Fluor 647	ThermoFisher	Cat#A27018; RRID:AB_2536082
LI-COR IRDye 680RD Goat anti-Mouse	ThermoFisher	Cat#NC0252290
Anti-rabbit IgG, HRP-linked Antibody	Cell Signaling Technology	Cat#7074; RRID:AB_2099233
Anti-mouse IgG, HRP-linked Antibody	Cell Signaling Technology	Cat#7076; RRID:AB_330924
Anti-beta Actin antibody	Abcam	Cat#ab8226; RRID:AB_306371
