# Supplementary material for: Myeloid Zfhx3 deficiency protects against hypercapnia-induced suppression of host defense against influenza A virus
Source: JCI Insight. 2024 Jan 16;9(4):e170316. doi: 10.1172/jci.insight.170316 (PMC11143927; doi:10.1172/jci.insight.170316)
Supplement: Unedited blot and gel images [file jciinsight-9-170316-s045.pptx]

## Slide 1
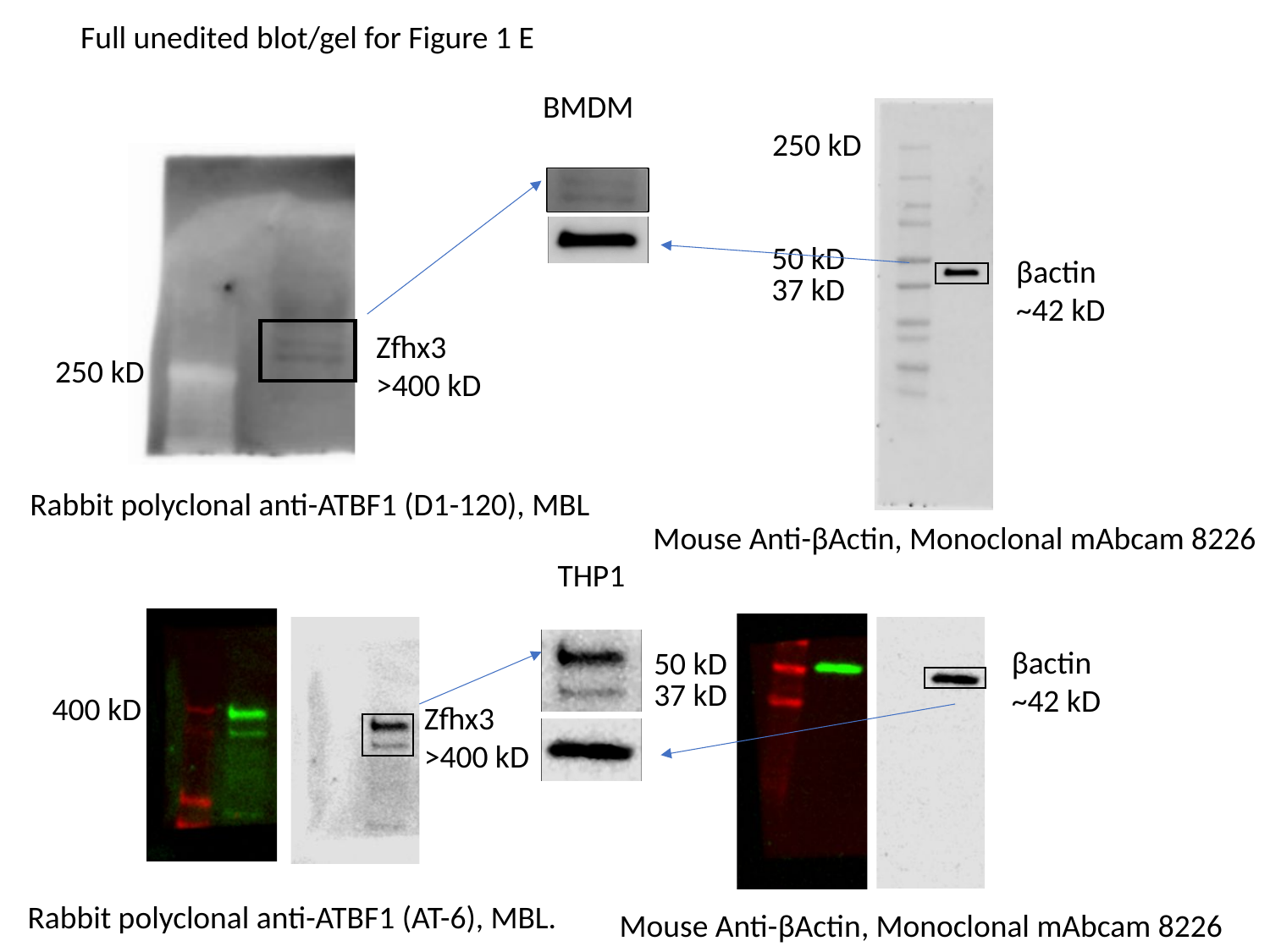

Full unedited blot/gel for Figure 1 E
BMDM
250 kD
50 kD
βactin
~42 kD
37 kD
Zfhx3
>400 kD
250 kD
Rabbit polyclonal anti-ATBF1 (D1-120), MBL
Mouse Anti-βActin, Monoclonal mAbcam 8226
THP1
βactin
~42 kD
50 kD
37 kD
400 kD
Zfhx3
>400 kD
Rabbit polyclonal anti-ATBF1 (AT-6), MBL.
Mouse Anti-βActin, Monoclonal mAbcam 8226
